# Production of NaOH-Modified Banana Pseudostem Biochars through Controlled Heating for Tetracycline Removal

**DOI:** 10.1021/acsomega.6c05829

**Published:** 2026-10-07

**Authors:** Maria Elisa Avila Barboza, João Antonio Tavares Barboza, Miriany Avelino Moreira Fernandez, Guilherme Max Dias Ferreira

**Affiliations:** † Group of Materials, Interfaces, and Solutions (MatIS), Department of Chemistry, Institute of Natural Sciences, 67739 Federal University of Lavras, Campus Universitário, Lavras, Minas Gerais 37203-202, Brazil; ‡ Department of Chemistry, Institute of Natural Sciences, Federal University of Lavras, Campus Universitário, Lavras, Minas Gerais 37203-202, Brazil

## Abstract

Proposing efficient adsorbents from agro-industrial waste for the removal of emerging contaminants, particularly tetracycline (TC), from aqueous systems remains a critical environmental challenge. In this study, we systematically investigate the influence of NaOH pretreatment combined with stepwise pyrolysis on the physicochemical properties and TC adsorption performance of biochars derived from banana pseudostem biomass. The biomass (with or without NaOH pretreatment) was subjected to pyrolysis at a heating rate of 15 °C min^–1^ under different thermal regimes, comprising either a single isothermal step at 400 °C or a stepwise heating pathway (215–400 °C), yielding unmodified and alkali-modified biochars for each condition. Physicochemical characterization (FTIR, XRD, BET, and pH_PZC_) revealed that alkaline treatment increased surface basicity, promoted the formation of oxygenated and carbonate phases, and improved pore development. The introduction of intermediate heating plateaus during pyrolysis also favored gradual devolatilization and structural rearrangement, contributing to the development of a more organized porous structure and greater accessibility of adsorption sites. Under unbuffered conditions at an initial pH of 6.0, the alkali-modified biochar produced through the 215–400 °C heating pathway exhibited an apparent adsorption capacity of 177.1 mg g^–1^. For the NaOH-modified biochars, kinetic data were better represented by the pseudo-second-order model, whereas equilibrium data were adequately described by Freundlich and Sips-type models, indicating heterogeneous adsorption behavior. The enhanced adsorption capacity was associated with the combined effects of surface chemistry, aromatic domains, and pore accessibility rather than with a single adsorption mechanism. These findings demonstrate that NaOH modification combined with controlled heating stages leads to the formation of biochars with enhanced TC adsorption capacity.

## Graphical abstract

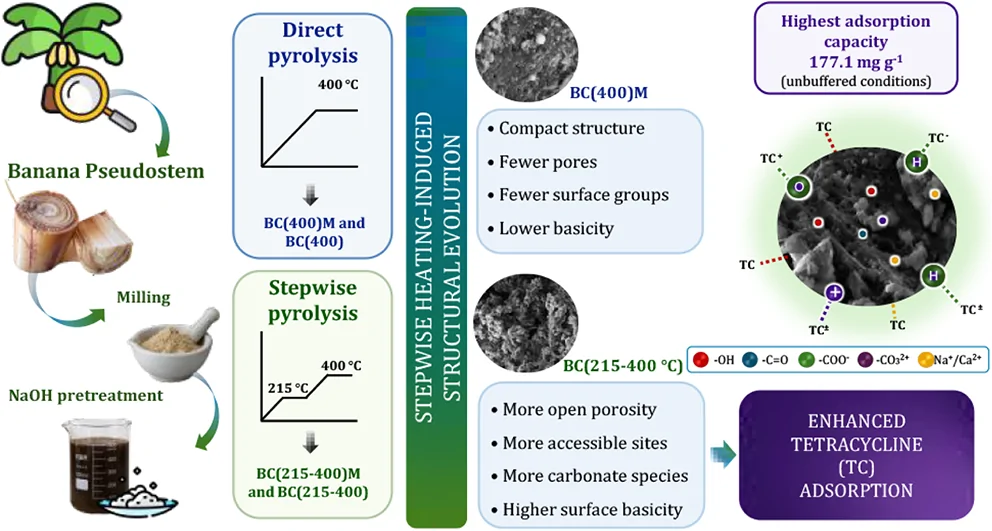

## 1 Introduction

Agroindustry is one of the fundamental pillars of the modern economy, supplying food, fibers, and bioenergy to society. However, the increasing volume of agro-industrial waste has raised significant environmental concerns, especially in tropical countries with intense agricultural activity, such as Brazil.^1^ Large quantities of waste are often improperly disposed of in soil or water bodies, contributing to environmental degradation and hindering sustainable reuse strategies.^2^ Most of these residues are lignocellulosic and primarily composed of cellulose, hemicellulose, and lignin, making them promising raw materials for conversion into higher value-added materials.^3^

Banana (*Musa* *spp.*) is one of the most relevant food crops globally, with substantial economic and social importance.^4^ In 2023, Brazil produced approximately 7 million tons of bananas, ranking as the fourth largest producer worldwide.^5^ However, this production generates significant volumes of waste. For every ton of bananas produced, approximately 100 kg of fruit are discarded, and around 4 tons of waste are generated, including 160 kg of stalks, 440 kg of peels, 480 kg of leaves, and 3 tons of pseudostem. The pseudostem represents over 80% of the plant’s biomass, and is commonly left in the field postharvest, where it can contribute to soil contamination, attract disease vectors, and release volatile compounds.^6,7^

As a result, extensive research has focused on valorizing banana residues to enhance their economic value and promote sustainable applications. Among these, the pseudostem has attracted particular attention and has been exploited through diverse technological routes, including its use in pulp production for the paper industry, the extraction of microcrystalline cellulose, and its application as a growth substrate for tolerant microalgae capable of efficiently removing arsenic from aqueous systems.^8–10^

In recent years, lignocellulosic residues have also gained attention as carbonaceous precursors for adsorptive materials such as biochars, which have shown potential for the removal of environmental contaminants from water matrices including dyes, toxic metals, and pharmaceuticals.^11^ Biochars are carbon-rich materials produced through the controlled thermal decomposition of biomass under an inert atmosphere, and have been extensively evaluated for environmental applications, including contaminant adsorption, soil remediation, and wastewater treatment.^12–14^ The physicochemical properties of these materials are heavily influenced by pyrolysis parameters, particularly temperature. Higher pyrolysis temperatures tend to increase pH, basic functional group content, and ash yield, while reducing acidic functionalities and volatile matter content.^13–15^

To enhance the adsorption properties of biochars, various modification strategies have been explored, including alkaline pretreatment of biomass with agents such as NaOH, and controlled thermal profiling during pyrolysis.^15^ Alkaline pretreatment facilitates surface oxidation by removing amorphous constituents, which promotes the formation of basic surface functional groups and increases surface area.^16^ Among alkaline reagents, NaOH and KOH are widely used, however, NaOH offers advantages such as lower required loading, reduced cost, decreased corrosivity, and better environmental compatibility.^17,18^ In parallel, controlling the thermal pathway, including the introduction of an intermediate pyrolysis stage, can alter the sequential decomposition of hemicellulose, cellulose, and lignin, thereby affecting pore development, surface chemistry, and the accessibility of adsorption sites.^19–22^ Despite these potential benefits, alkaline pretreatment and thermal-profile modulation have generally been investigated as separate strategies. Consequently, limited information is available on how NaOH pretreatment interacts with direct versus two-step pyrolysis to govern the physicochemical properties and adsorption behavior of biochars.

The adsorption of pharmaceutical residues using biochars is a topic of growing interest due to the widespread detection of such contaminants in natural waters.^23^ Antibiotics such as tetracycline (TC) are of particular concern. Following human or veterinary administration, 30 to 90% of the active compound can be excreted in unchanged form, accumulating in the environment.^24,25^ TC is highly soluble, poorly biodegradable, and capable of fostering antimicrobial resistance. These characteristics render its presence in aquatic environments a critical issue for public health and ecological stability.^26^ An investigation conducted at seven wastewater treatment facilities in Wisconsin (USA) revealed that TC ranked among the most found antibiotics, being detected in 80% of the samples analyzed.^27^ Moreover, TC can exist in anionic, cationic, or zwitterionic forms depending on pH, which significantly affects its interaction with adsorbent surfaces.^28^

Recent investigations emphasize the role of precursor composition, activation strategies, and surface chemistry in tuning the adsorption capacity of biochars for TC. A calcium-rich composite biochar derived from crayfish shells, prepared at 700 °C, achieved a high theoretical adsorption capacity of 168.53 mg g^–1^, attributed to the presence of CaCO_3_, developed porosity, and multiple binding mechanisms such as hydrogen bonding, π–π interactions, and metalorganic complexation.^29^ Another study demonstrated that lignin-derived biochar obtained from black liquor, pyrolyzed at 800 °C, reached a maximum TC adsorption capacity of 243.91 mg g^–1^, with performance governed by pore filling, electrostatic interaction, and hydrogen bonding.^30^ Meanwhile, a biochar synthesized from starch via dual activation with ZnCl_2_/LiCl and H_2_O_2_ exhibited a remarkably high adsorption capacity of 1357.9 mg g^–1^. This performance was attributed to its large surface area (1790 m^2^ g^–1^), elevated oxygen content (26%), and the synergistic contribution of oxygen-containing groups.^31^ Additionally, a composite material produced by anchoring porous zerovalent iron on distillers’ grains biochar demonstrated a high TC removal percentage (92.15%) combined with catalytic degradation activity, with reusability and stability in saline media, highlighting its potential for complex wastewater treatment scenarios.^32^

Although substantial progress has been made in understanding TC adsorption by alkali-modified biochars, limited information is available on how different pyrolysis pathways influence the physicochemical properties and adsorption performance of NaOH-pretreated banana pseudostem-derived biochars. Using a fixed NaOH pretreatment condition, this study tests the hypothesis that distinct pyrolysis strategies produce biochars with different structural, chemical, and textural properties, which, in turn, govern their TC adsorption performance.

## 2 Materials and Methods

### 2.1 Reagents

The reagents used in this study are listed in Table 1. All reagents were used without prior purification. Deionized water was employed for the preparation of all solutions.

**Table 1:** List of Reagents

Reagent	Formula	Purity (w/w)	Supplier
Tetracycline hydrochloride	C_22_H_24_N_2_O_8_ (HCl)	98.6%	Sigma-Aldrich
Hydrochloric acid	HCl	37.2%	Sigma-Aldrich
Sodium hydroxide	NaOH	98%	Synth
Phosphoric acid	H_3_PO_4_	85%	Synth
Acetic acid	H_3_CCOOH	99.7%	Synth
Boric acid	H_3_BO_3_	99.5%	Synth
Sodium chloride	NaCl	99.8%	Synth
Potassium phosphate monobasic	KH_2_PO_4_	100%	Proquimios
Calcium chloride	CaCl_2_	76%	Proquimios
Magnesium sulfate	MgSO_4_	100%	Dinâmica
Ethanol	C_2_H_6_O	99.2%	Êxodo Científca

### 2.2 Biomass Preparation and Alkaline Pretreatment

Banana pseudostem residues (*Musa* *spp.*) were collected from a rural property in Caldas, Minas Gerais, Brazil. The biomass was initially cut into ∼3 cm segments and thoroughly washed with running tap water followed by deionized water to remove surface-adhered solids and soluble compounds. The material was then placed in a ceramic tray and oven-dried at 60 °C for 72 h in an oven. After drying, the biomass was ground and sieved to obtain particles with size between 0.15 mm and 0.25 mm.

The alkaline pretreatment was carried out using a NaOH solution prepared to provide approximately 0.1 g NaOH per gram of dry biomass. Specifically, 20 g of biomass were treated with 250 mL of a 0.2 mol L^–1^ NaOH solution (0.05 mol NaOH), corresponding to a solid-to-liquid ratio of 1:12.5 (w/v), following alkaline modification strategies reported to produce biomass-derived adsorbents.^33^ The mixture was kept under static contact for 20 h at room temperature. After pretreatment, the material was dried at 60 °C for 24 h prior to pyrolysis.

### 2.3 Determination of Pyrolysis Temperatures by Thermogravimetric Analysis (TGA)

The raw banana pseudostem biomass was subjected to thermogravimetric analysis (TGA) to identify the temperature ranges associated with mass loss and the maximum degradation rates of the material’s lignocellulosic fractions. The TGA was conducted using a under a nitrogen atmosphere at a flow rate of 50 mL min^–1^. Approximately 5 mg of sample was heated from 25 to 900 °C at a heating rate of 10 °C min^–1^. Pyrolysis temperatures were defined based on the thermal degradation profile obtained from the TGA curve.

### 2.4 Biochar Production

Both untreated and NaOH-pretreated biomasses were subjected to pyrolysis in an EDG 3000 3P muffle furnace, at a heating rate of 15 °C min^–1^ and residence time of 1 h at the final (or intermediate) temperature. The following heating procedures were adopted: (i) the biomasses were directly pyrolyzed to 400 °C, yielding the unmodified biochar BC(400) and its NaOH-modified counterpart BC(400)­M; and (ii) the biomasses were heated and first held at an intermediate temperature of 215 °C (defined by TGA), then heated to 400 °C, producing BC(215–400) and BC(215–400)­M. The suffix “M” in the sample codes indicates materials that underwent alkaline pretreatment with NaOH prior to pyrolysis. Figure S1 illustrates a schematic representation of the production process.

After carbonization, all biochars were manually ground and sieved to obtain particles smaller than 0.25 mm. The biochars were intentionally not washed after pyrolysis to evaluate the resulting materials in their as-produced form, considering both the carbonaceous matrix and the retained inorganic species, while avoiding additional water consumption, wastewater generation, and processing steps. Experiments performed with washed materials are described in Section 2.6.7.

### 2.5 Biochar Characterization

#### 2.5.1 Fourier Transform Infrared Spectroscopy (FTIR)

The surface functional groups of the biochars were analyzed using attenuated total reflectance Fourier transform infrared spectroscopy (ATR-FTIR). Spectra were recorded on a Varian 600-IR Series spectrometer in ATR mode, covering the spectral range of 400–4000 cm^–1^, with a resolution of 4 cm^–1^ and a total of 32 scans per sample.

#### 2.5.2 Scanning Electron Microscopy and Energy-Dispersive X-ray Spectroscopy (SEM–EDS)

The morphological characterization of the biochars was performed by scanning electron microscopy (SEM) using an Ultra-High-Resolution Scanning Electron Microscope Tescan Clara-UHR. Elemental composition was determined by energy-dispersive X-ray spectroscopy (EDS) using a Scanning Electron Microscope Leo EVO 40 XVP Carl Zeiss coupled to a Bruker Quantax EDX detector. Prior to imaging, a small quantity of each sample was mounted onto aluminum stubs using carbon adhesive tape and subsequently coated with a thin carbon layer. The samples were stored under vacuum until analysis. Micrographs were acquired at various magnifications to assess the morphological and chemical composition of the biochars.

#### 2.5.3 Thermogravimetric Analysis (TGA)

The TGA of the biochars was conducted using a DTG-60*A*/60AH (Shimadzu) under a nitrogen atmosphere at a flow rate of 50 mL min^–1^. Approximately 5 mg of sample was heated from 25 to 900 °C at a heating rate of 10 °C min^–1^.

#### 2.5.4 X-ray Diffraction (XRD)

X-ray diffraction (XRD) analyses were performed using a Bruker D2 PHASER (Bruker, USA). The measurements were carried out in continuous scan mode over a 2θ range of 5° to 70°, with an angular step size of 0.2021°, resulting in 3217 steps. The counting time per step was 0.6 s.

#### 2.5.5 Surface Area and Porosity Analysis

N_2_ adsorption–desorption isotherms were performed using a Quantachrome NOVA 600 surface area and pore size analyzer, at 77 K. Prior to the measurements, the samples were subjected to vacuum degassing at 120 °C for 5 h to remove physiosorbed species. The specific surface area (*S*_BET_) was determined using the Brunauer–Emmett–Teller (BET) method. The micropore surface area and micropore volume were calculated by the t-plot method, while the mesopore size distribution and average pore diameter were obtained using the Barrett–Joyner–Halenda (BJH) method applied to the desorption branch of the isotherm.^69^

#### 2.5.6 Number of Acidic and Basic Functions

The determination of the number of acidic and basic functional groups was determined via conductometric titration. Approximately 150 mg of each biochar was suspended in 70.0 mL of a 0.035 mol L^–1^ HCl aqueous standard solution and placed in a jacketed titration cell connected to an ultrathermostatic water bath (LUCADEMA), maintained at 25.0 ± 0.5 °C under constant magnetic stirring until stabilization of the conductivity. The dispersion was then titrated with a 0.198 mol L^–1^ NaOH solution, added in sequential increments of 0.300 or 0.150 mL. After each addition, the electrical conductivity was measured. Experimental values were corrected for dilution effects according to eq 1:

1 $$k_{corrected}=k\frac{\left(V_{i}+V\right)}{V}$$

where *k* and *k*_*corrected*_ are the experimental and dilution-corrected conductivity values (μS cm^–1^), *V*_*i*_ is the initial solution volume (mL), and *V* is the cumulative volume of NaOH added.

The amount of basic functional groups was calculated using eq 2:

2 $$n_{bf}=C_{NaOH}\frac{\left(V_{0}-V_{1}\right)}{m_{bc}}$$

where *C*_*NaOH*_ is the titrant concentration (mol L^–1^), *m*_*bc*_ is the mass of biochar used (g), *V*_*0*_ is the NaOH volume at the first inflection point of the blank experiment, and *V*_*1*_ is the corresponding volume in the sample titration. Results are expressed in mmol g^–1^.

The second linear region of the conductivity curve was used to estimate the concentration of acidic surface groups, calculated by eq 3:

3 $$n_{af}=C_{NaOH}\frac{\left(V_{2}-V_{1}\right)}{m_{bc}}$$

where *V*_2_ is the NaOH volume at the intersection of the second and third linear regions of the titration curve. All other parameters are as previously defined.

#### 2.5.7 pH at the Point of Zero Charge (pH_PZC_)

The point of zero charge (pH_PZC_) was determined by the solid addition method. For each sample, 20.0 mg of biochar was added to 20.00 mL of a 0.100 mol L^–1^ NaCl aqueous solution, whose initial pH (pH_0_) was adjusted from 2 to 12 using 0.1 mol L^–1^ HCl or NaOH. The suspensions were agitated in a shaker incubator (model NT715, Nova Técnica, Brazil) at 25 ± 1 °C for 24 h. After equilibration, the final pH (pH_f_) was measured using a bench pH meter (model mPA210, MS Tecnopon, Brazil), and the pH change (ΔpH) was plotted as a function of the initial pH (pH_0_). The pH_PZC_ was defined as the pH value for which ΔpH = 0. All experiments were conducted in duplicate.

### 2.6 Adsorption Experiments

#### 2.6.1 Initial Evaluation of Adsorptive Capacity

To identify the most efficient pyrolysis strategy, preliminary adsorption tests were performed using approximately 10 mg of each biochar dispersed in 10.0 mL of a 100 mg L^–1^ TC solution, without previous pH adjustment. The dispersions were agitated at 120 rpm for 24 h. After equilibrium, the samples were centrifuged, and the supernatant was analyzed by UV–visible spectrophotometry in the ultraviolet region at 276 nm (model AJX-3000 PC, AJMICRONAL). The final pH of each sample was measured.

Experiments were conducted in triplicate, and results are reported as the mean values. The amount of TC adsorbed per unit mass of adsorbent (*q_e_*), in mg g^–1^, was calculated using eq 4:

4 $$q_{e}=\frac{\left(C_{i}-C_{e}\right)}{m}V$$

where *C_i_* and *C_e_* are the initial and equilibrium concentrations of TC (mg L^–1^), *V* is the volume of solution (L), and *m* is the mass of biochar (g).

The removal efficiency (%R) was determined using eq 5:

5 $$\%R=\frac{C_{i}-C_{f}}{C_{i}}\times 100$$

where *C*_f_ and *C*_*i*_ represent the final and initial concentrations of TC (mg L^–1^), respectively.

#### 2.6.2 Buffer Solution Preparation

Because contact with the biochars increased the solution pH and consequently altered the ionic speciation of TC, the experiments investigating the effect of pH on TC adsorption, as well as the kinetic and selected equilibrium studies (Sections 2.6.3 and 2.6.4), were conducted in buffered media to attenuate pH variations during the adsorption process. Britton–Robinson (B–R) buffer solutions were prepared using a mixture of phosphoric, boric, and acetic acids, each at a final concentration of 0.01 mol L^–1^.

#### 2.6.3 Effect of pH on TC Adsorption

To assess the influence of pH on TC adsorption, approximately 10 mg of either BC(400)­M or BC(215–400)­M was added to 10.00 mL of a 50.0 mg L^–1^ TC solution prepared in Britton–Robinson buffer, with the pH adjusted to approximately 4.0, 6.0, 8.0, or 10.0 using NaOH or HCl solutions. Adsorption experiments were carried out in triplicate. After 24 h of contact time, the samples were centrifuged, and the supernatant was analyzed by UV–visible spectrophotometry in the ultraviolet region at 275, 276, 274, and 271 nm for pH 4.0, 6.0, 8.0, and 10.0, respectively. Values of *q_e_* and %R were obtained as described in Section 2.6.1.

#### 2.6.4 Kinetic and Equilibrium Studies

For the kinetic assays, approximately 10 mg of each biochar was dispersed in 10.0 mL of a 50.0 mg L^–1^ TC solution buffered with B-R buffer at initial pH of approximately 6.0. The suspensions were maintained at 25 ± 1 °C under constant agitation (120 rpm) in a shaker incubator for predetermined intervals ranging from 10 to 2160 min. Subsequently, the supernatants were collected, centrifuged and analyzed using UV–visible spectrophotometry, as described in Section 2.6.1. The nonlinear models of Lagergren pseudo-first order, pseudo-second order, and intraparticle diffusion were evaluated (Table 2, eqs 6–8).

**Table 2:** List of Kinetic and Adsorption Isotherm Models in Integrated Form

	Model	Equation	Reference
Kinetics	Pseudo-first order	6 $q_{t}=q_{e}\left(1-e^{-k_{1}t}\right)$	^34^
	Pseudo-second order	$q_{t}=\frac{{q_{e}}^{2}k_{2}t}{1+q_{e}k_{2}t}$ 7	^35^
	Intraparticle diffusion	8 $q_{t}=k_{dif}t^{1/2}+C$	^36^
Isotherms	Freundlich	9 $q_{e}=K_{F}C_{e}^{1/n}$	^37^
	Langmuir	10 $q_{e}=\frac{q_{max}K_{L}C_{e}}{1+K_{L}C_{e}}$	^38^
	Sips	11 $q_{e}=\frac{q_{max}{\left(K_{S}C_{e}\right)}^{n_{S}}}{1+{\left(K_{S}C_{e}\right)}^{n_{S}}}$	^39^

Equilibrium adsorption isotherms were obtained by mixing approximately 10 mg of biochar with 10.0 mL of TC solutions of varying initial concentrations (10.0–400 mg L^–1^), prepared either with or without buffer, with the pH adjusted to approximately 6.0 prior to the adsorption experiments. The suspensions were agitated at 25 ± 1 °C for 36 h at 120 rpm. Subsequently, the supernatants were centrifuged, diluted when necessary, and analyzed in a UV–visible spectrophotometer at 276 nm. For the system without buffer, the analytical curves were prepared at the final pH of the samples after adsorption.

To model the adsorption equilibrium, the nonlinear forms of the Freundlich, Langmuir, and Sips isotherms were evaluated (Table 2, eqs 9–11).

#### 2.6.5 Effect of Electrolyte Type on TC Adsorption

The influence of different electrolytes on TC adsorption was investigated using aqueous solutions containing 0.1 mol L^–1^ KH_2_PO_4_, CaCl_2_, or MgSO_4_. TC solutions (100 mg L^–1^) were prepared in each electrolyte solution, and the initial pH was adjusted to approximately 6.0. Subsequently, approximately 10 mg of BC(400)­M or BC(215–400)­M was added to 10.0 mL of the corresponding TC solution. The suspensions were agitated at 120 rpm and 25 ± 1 °C for 24 h. After adsorption, the final pH was measured, the suspensions were centrifuged, and the residual TC concentration was determined according to the procedure described in Section 2.6.1. Calibration curves were prepared using the corresponding electrolyte solutions to minimize matrix effects during UV–Vis analysis. All experiments were performed in triplicate.

#### 2.6.6 Adsorption Performance in Tap Water

The adsorption performance of the biochars was also evaluated using tap water collected at the Federal University of Lavras (Minas Gerais, Brazil). The initial pH, electrical conductivity, and turbidity of the tap water were measured using an MS Tecnopon mPA210 pH meter, an MS Tecnopon conductivity meter, and a Hanna Instruments HI98703-02 turbidimeter, respectively. A TC solution (100 mg L^–1^) was prepared using tap water, and approximately 10 mg of BC(400)­M or BC(215–400)­M was added to 10.0 mL of the solution. The suspensions were agitated at 120 rpm and 25 ± 1 °C for 24 h. After adsorption, the final pH was measured, and the residual TC concentration was determined according to the procedure described in Section 2.6.1. All experiments were performed in triplicate.

#### 2.6.7 Effect of Postpyrolysis Washing on TC Adsorption

To evaluate the influence of residual inorganic species on TC adsorption, the modified biochars BC(400)­M and BC(215–400)­M were washed with deionized water until the supernatant pH reached approximately 7.0. The washed materials were designated BW(400)­M and BW(215–400)­M, where “W” denotes that the material was washed. Adsorption experiments were conducted by contacting approximately 10 mg of each biochar with 10.0 mL of a 100 mg L^–1^ TC solution at an initial pH of 6.0 under both buffered and unbuffered conditions. The suspensions were agitated at 120 rpm and 25 °C for 24 h. After equilibrium, TC concentrations were determined according to the procedure described in Section 2.6.1. All experiments were performed in triplicate.

#### 2.6.8 Leaching of Inorganic Species from the Biochars

The release of inorganic species from BC(400)­M, BC(215–400)­M and BW(215–400)­M during adsorption was evaluated. Briefly, approximately 10 mg of each biochar was contacted with 10.0 mL of a 50 mg L^–1^ TC solution at an initial pH of 6.0. The suspensions were agitated at 120 rpm and 25 ± 1 °C for 24 h. Control experiments were conducted under identical conditions using deionized water adjusted to initial pH of 6.0 instead of the TC solution to evaluate the leaching of inorganic species in the absence of TC. After equilibration, the suspensions were centrifuged, and the supernatants were collected for elemental analysis. Ca and Mg concentrations were determined by flame atomic absorption spectrometry (FAAS), whereas Na and K concentrations were determined by flame photometry. All experiments were performed in triplicate.

#### 2.6.9 Regeneration and Reusability of the Biochar

Adsorption–desorption experiments were conducted to evaluate the regeneration and reusability of the biochars. For the adsorption step, approximately 10 mg of BC(400)­M or BC(215–400)­M was added to 10.0 mL of a 50 mg L^–1^ TC solution, with an initial pH adjusted to approximately 6.0. The suspensions were agitated at 120 rpm for 24 h at 25 ± 1 °C. After adsorption, the suspensions were centrifuged, and the supernatants were collected for TC quantification according to the procedure described in Section 2.6.1.

For the desorption step, the recovered biochars were contacted with 10.0 mL of 99.2% ethanol and agitated at 120 rpm and 25 °C for 2 h. The biochars were subsequently recovered, rinsed with deionized water, and dried before being reused in the next adsorption cycle under the same experimental conditions. Three consecutive adsorption cycles were performed, and all experiments were conducted in triplicate.

#### 2.6.10 Postadsorption Characterization

BC­(215–400)­M samples were characterized after contact with deionized water, used as a process control, and after TC adsorption at initial concentrations of 50 or 400 mg L^–1^. The experiments were conducted at an initial pH of 6.0 (unbuffered) using an adsorbent dose of 1.0 g L^–1^ with a contact time of 24 h at 25 ± 1 °C and 120 rpm. After contact, the solids were recovered by filtration and washed three times with 5.0 mL of deionized water per cycle, corresponding to a total washing volume of 15 mL. The samples were subsequently dried at 70 °C for 24 h before characterization by ATR-FTIR spectroscopy, thermogravimetric analysis, and elemental analysis.

Spectra were obtained by attenuated total reflectance Fourier-transform infrared spectroscopy (ATR-FTIR) using a Varian 600-IR Series spectrometer. The spectra were recorded over the range of 400–4000 cm^–1^ at a resolution of 2 cm^–1^ using 32 scans per sample.

Carbon and nitrogen contents were determined by elemental analysis coupled with isotope-ratio mass spectrometry (EA–IRMS). A Thermo Fisher Scientific Delta V Advantage isotope-ratio mass spectrometer equipped with a ConFlo IV universal interface and a peripheral elemental analyzer was used. The system was operated using ISODAT software, and approximately 2 mg of each sample was analyzed.

Thermogravimetric analysis was performed using a DTG-60*A*/60AH (Shimadzu) under a nitrogen atmosphere at a flow rate of 50 mL min^–1^. Approximately 5 mg of each sample was heated from 25 to 600 °C at a heating rate of 10 °C min^–1^.

## 3 Results

### 3.1 Fourier Transform Infrared Spectroscopy (FTIR)

The FTIR spectra (Figure 1) reveal the structural changes in the raw biomass and the biochars produced under different pyrolysis conditions, with and without NaOH pretreatment.

**Figure 1:**
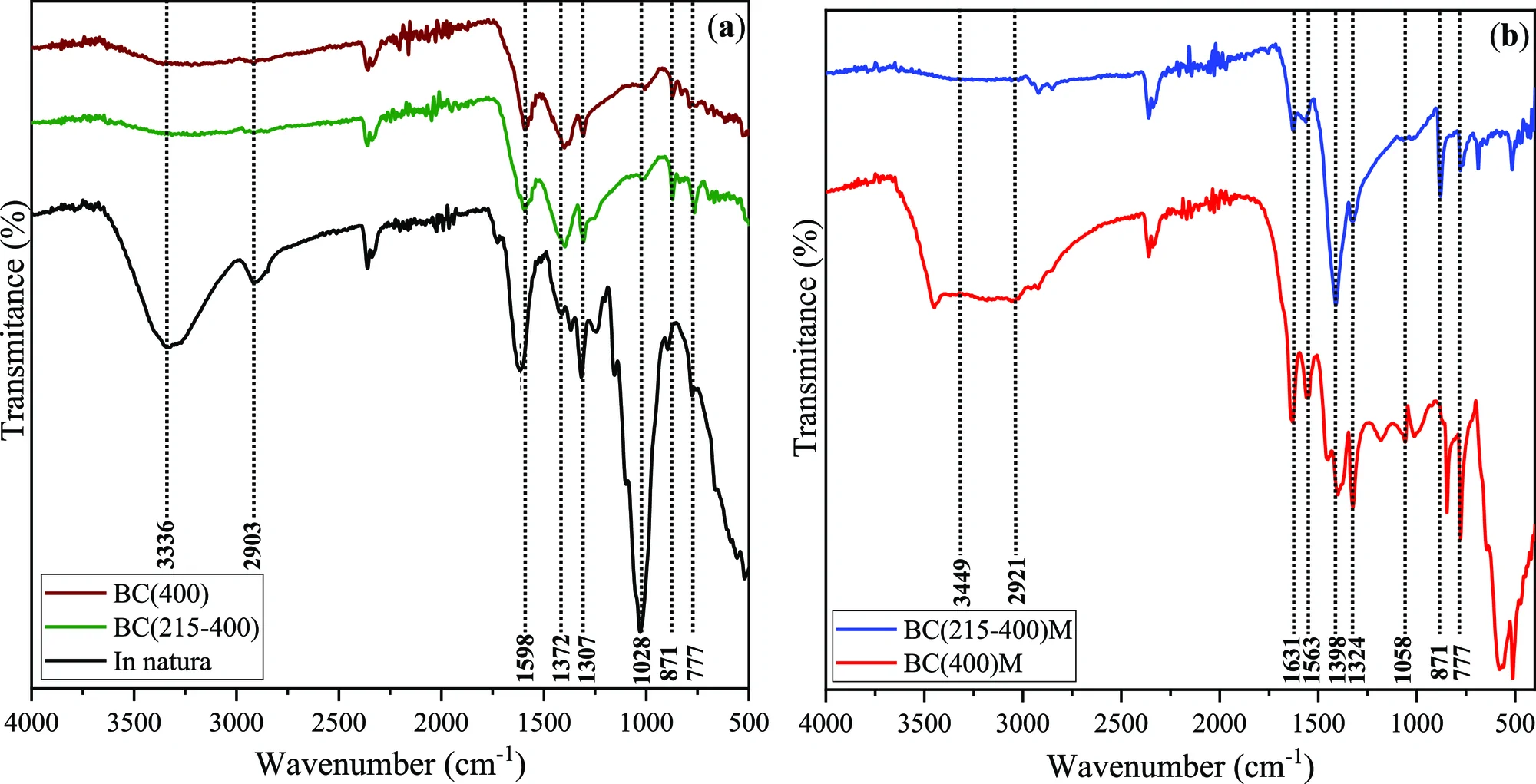
FTIR spectra of the materials: (a) BC(400), BC(215–400), and untreated biomass; and (b) NaOH-modified biochars.

The untreated biomass exhibited a broad band centered at 3336 cm^–1^, assigned to O–H stretching vibrations of hydroxyl groups in cellulose and hemicellulose.^40^ After pyrolysis, this band disappeared in all samples except for BC(400)­M (3750–2500 cm^–1^), indicating partial preservation of hydroxyl functionalities, likely stabilized by the alkaline medium. For BC(400)­M, the broadening of the band with a clear maximum at 3349 cm^–1^ indicates a higher surface heterogeneity, likely associated with enhanced hydrogen bonding interactions.

The aliphatic C–H asymmetric and symmetric stretching vibrations, near 2921 and 2850 cm^–1^, were detected in BC(215–400)­M and BC(400)­M, confirming the presence of residual −CH_2_ and −CH_3_ groups from lignocellulosic structures.^41,42^

The sharp carbonyl (C=O) stretching band at 1728 cm^–1^, prominent in the raw biomass, was not detected in any biochar sample, indicating the thermal decomposition of carboxylic, ester, and ketonic groups during pyrolysis. In contrast, bands associated mainly with aromatic C=C stretching were observed at 1631 cm^–1^ and 1563 cm^–1^ for BC(400)­M and BC(215–400)­M, and at 1598 cm^–1^ for BC(400) and BC­(215-400).^43,44^ The shift in band position after NaOH pretreatment suggests changes in the local chemical environment of these aromatic structures.

Bands in the 1550–1598 cm^–1^ region can also contain contributions from the asymmetric stretching of carboxylate (−COO^–^) or carbonate (CO_3_^2–^) groups, and appeared in all pyrolyzed samples, particularly those treated with NaOH. The presence of these bands in the modified materials can suggest base-induced oxidation and mineralization processes.^45,46^ XRD analysis confirmed the presence of trona (Na_3_H­(CO_3_)_2_·2H_2_O) in alkali-modified samples, which supports the attribution of these bands (see Section 3.4). Trona typically exhibits IR features near 1590 cm^–1^, 1398–1409 cm^–1^ and bending modes in the 871–777 cm^–1^ region.^47,48^

An increase in band intensity was observed in BC(215–400)­M spectrum at 1398 cm^–1^ and in BC(400)­M spectrum at 1409 cm^–1^. This region can be associated with symmetric stretching of carbonate groups and/or skeletal vibrations of C=C bonds, reflecting both mineral presence and structural rearrangements in aromatic domains.^49^

The band in the 1315–1325 cm^–1^ range, attributed to C–H bending or internal vibrations of carbonate-containing minerals such as trona, was prominent in BC(215–400)­M (1325 cm^–1^) and BC(400)­M (1324 cm^–1^). The C–O–C stretching vibration associated with polysaccharides in cellulose and hemicellulose appeared at 1031 cm^–1^ in the untreated biomass and was retained only in BC(400)­M, slightly shifted to 1011 cm^–1^, suggesting limited preservation of glycosidic linkages under milder pyrolysis conditions. In all other biochars, this band was absent, suggesting complete depolymerization of carbohydrate structures.^11,40^

Finally, all biochars displayed bands in the 871–777 cm^–1^ region, attributed to out-of-plane bending vibrations of aromatic C–H bonds. These signals confirm the aromatization of the carbon matrix and overlap with carbonate-related bands, reinforcing the coexistence of aromatic and mineral structures on the biochar surfaces.^50^

### 3.2 Scanning Electron Microscopy and Energy-Dispersive X-ray Spectroscopy (SEM–EDS)

Figure 2 shows the SEM images highlighting the structural changes of the biochars with thermal treatment and NaOH modification.

**Figure 2:**
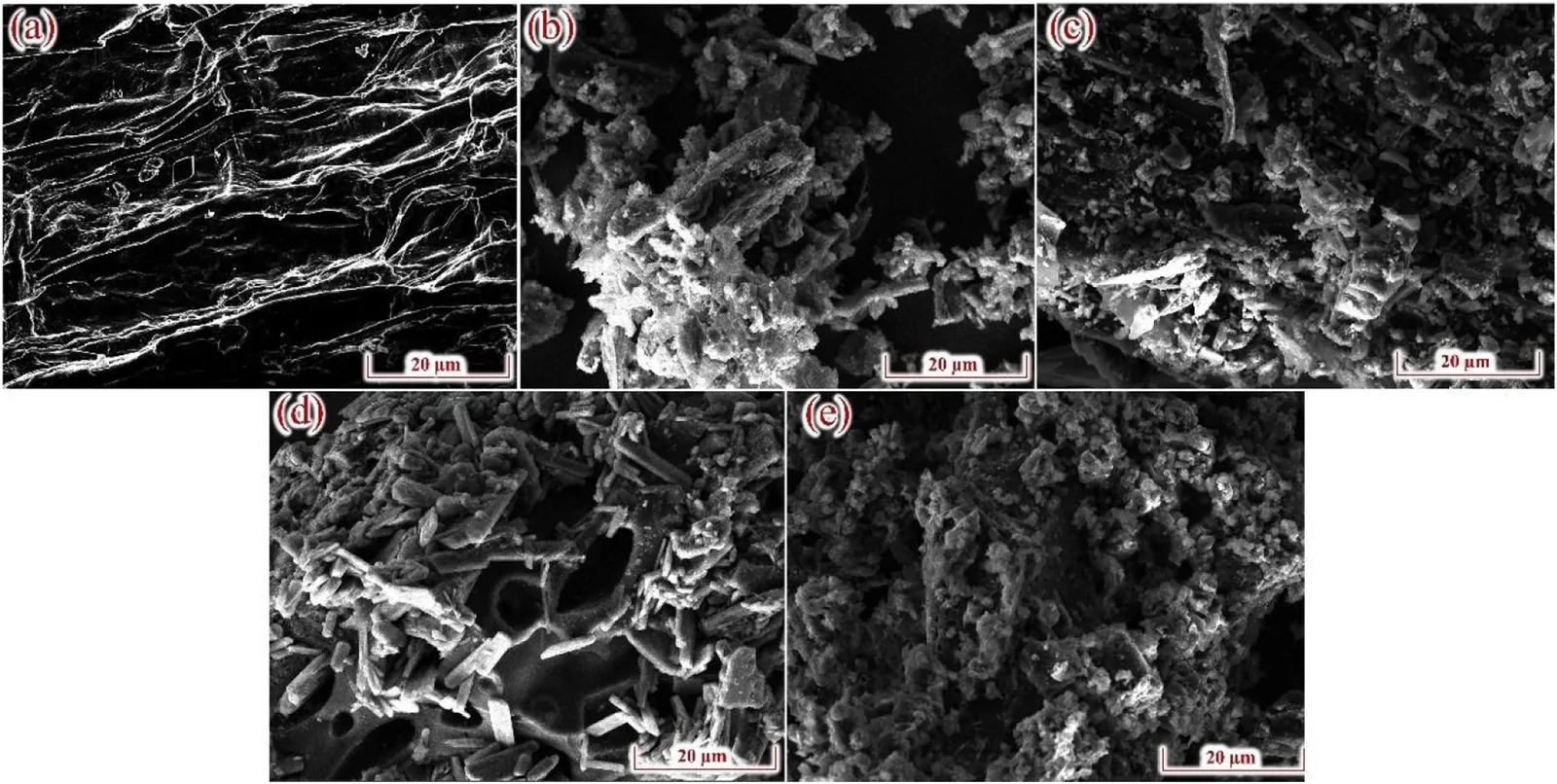
SEM images for raw material and biochars (a) untreated biomass, (b) BC400, (c) BC(215–400), (d) BC(400)­M, (e) BC(215–400)­M.

The micrographs in Figure 2b–c for BC(400) and BC(215–400) (see also Figures S3–S4 in Supporting Information) exhibit compact, fibrous structures with limited surface disruption and no evident pore formation. The morphology is predominantly rigid, with aligned filaments and low textural complexity. Using NaOH treatment, morphological changes are observed (Figure 2d–e and Figures S5–S6). BC(400)­M presents a more fragmented and irregular surface, marked by the appearance of open channels and surface agglomerates. These features suggest localized disruption of the carbon matrix. Similarly, BC(215–400)­M exhibits a flake-like, flocculated structure with visible cavities and enhanced surface roughness. Such differences are likely associated with the different thermal histories applied to the NaOH-impregnated biomass. The intermediate holding step at 215 °C may modify the decomposition sequence of lignocellulosic components before the final carbonization stage, leading to a distinct evolution of the carbon framework. The gradual release of volatile compounds during this intermediate stage may also promote the progressive formation and interconnection of pores of different dimensions, resulting in a more heterogeneous porous architecture and, consequently, the markedly different morphologies observed after pyrolysis.^51^

EDS results (Table S1) reveal that oxygen and potassium are predominant in all samples, consistent with the elemental profile of plant-derived biochars.^52^ Na appears exclusively in the modified materials, consistent with the NaOH incorporation during pretreatment. In contrast, unmodified biochars retained minor amounts of Al, Si, and Mg, possibly inherited from the biomass or soil impurities.

The increase in surface oxygen content in NaOH-modified samples supports the presence of oxygenated functional groups, also indicated by FTIR. These groups may contribute to TC adsorption via hydrogen bonding or electrostatic interactions. Additionally, alkali and alkaline earth metals (K, Ca, Na) are known to influence adsorption behavior by enabling cation exchange or interacting with charged moieties of the target contaminant.^53,54^

### 3.3 Thermogravimetric Analysis (TGA)

Thermogravimetric analysis (TGA) (Figure 3a–e) was employed to assess the thermal degradation behavior of banana pseudostem biomass and its derived biochars.

**Figure 3:**
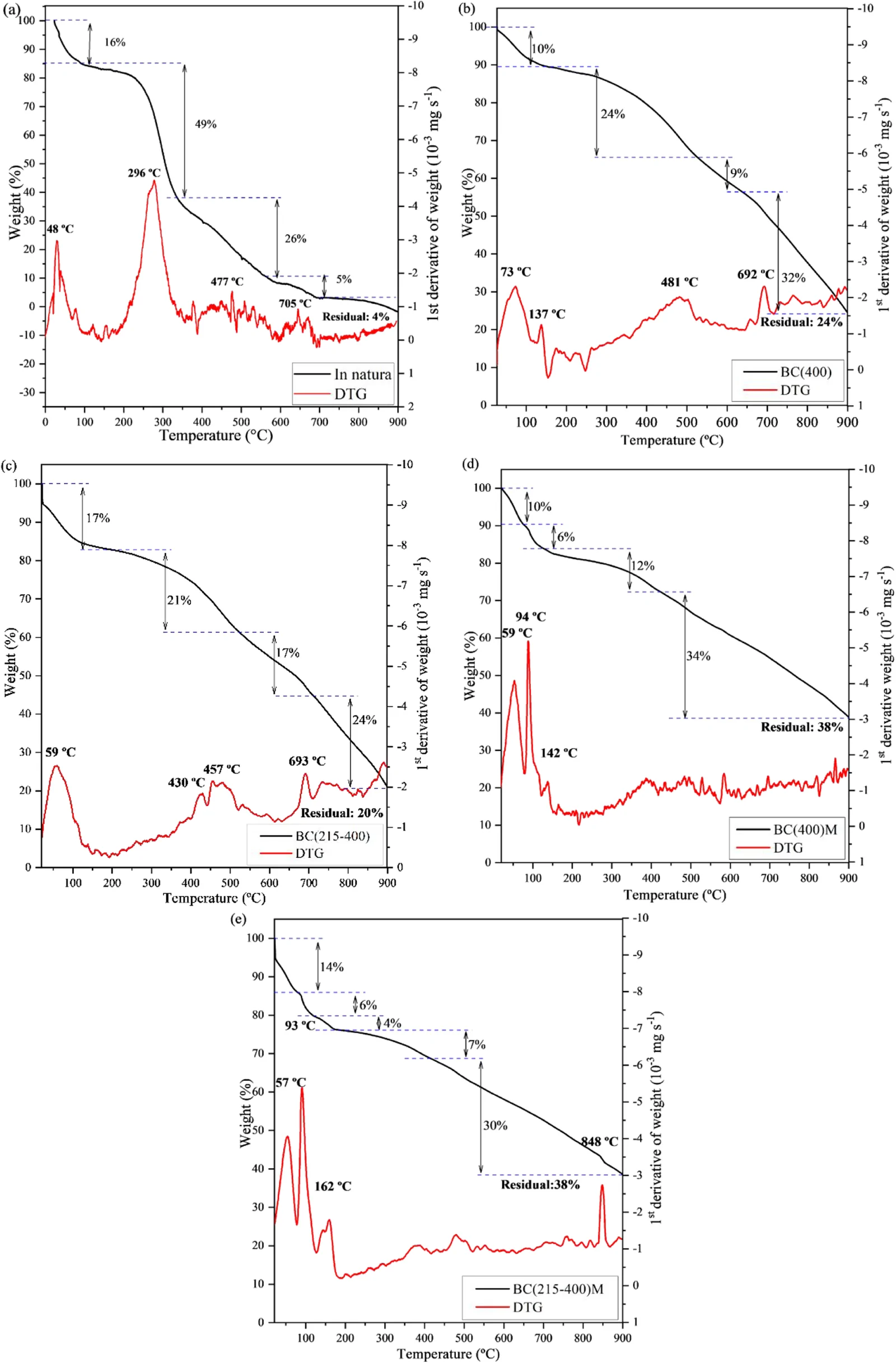
TGA and DTG curves of raw material (a) in natura and biochars (b) BC400, (c) BC(215–400), (d) BC(400)­M, and (e) BC(215–400)­M.

The in natura biomass exhibited four distinct stages of mass loss. The first event occurred below 152 °C, with 16% mass loss and a DTG peak at 48 °C, corresponding to moisture evaporation.^55^ Between 152 and 346 °C, the material lost 49% of its mass, attributed to the decomposition of hemicellulose and partial cellulose degradation, with a maximum rate at 296 °C. From 346 to 600 °C, an additional 26% mass loss was observed, mainly associated with advanced cellulose degradation and progressive lignin decomposition, with a DTG peak around 477 °C.^56,57^ The total residual mass was 4%, confirming the high volatile content, typical of lignocellulosic biomass.

Based on this decomposition profile, 215 °C was selected as the intermediate temperature for BC­(215-400) and BC­(215-400)­M production because it corresponds to the onset of hemicellulose decomposition, while remaining below the temperature range of extensive cellulose degradation. Maintaining the biomass at this temperature before heating to 400 °C was intended to promote a more gradual release of volatile compounds and reduce the overlap between the thermal degradation of the main lignocellulosic components. Subsequently, 400 °C was selected to ensure further cellulose decomposition and biochar formation. This stepwise heating strategy was adopted to enable progressive volatilization, preserve lignin-rich domains, and induce structural reorganization conducive to carbonization and microporosity development.^58^

BC­(400) exhibited reduced mass loss (75%) and a residual mass of 24% at 900 °C. Its main DTG peak occurred at 481 °C, probably associated with the persistence of lignin-derived structures.^59^ The stepwise-pyrolyzed biochar, BC(215–400), presented DTG peaks at 430 and 457 °C, which can be ascribed to overlapping degradation of partially stabilized lignin and residual cellulose.^60^ Its final residue was 20%, suggesting the formation of a less carbon-rich and thermally stable structure than BC(400).

NaOH-modified biochars showed further enhancement in thermal stability. BC(400)­M presented initial DTG peaks at 59 and 94 °C, likely reflecting water loss and other volatile species.^61^ Its major mass loss occurred beyond 500 °C, with a residual mass of 38% at 900 °C. BC(215–400)­M showed similar early thermal events and a significant DTG peak at 848 °C, which may be associated with the thermal decomposition or transformation of inorganic carbonate phases identified by XRD.^61,62^

These observations support that NaOH modification facilitates the cleavage of ether and ester linkages and promotes dehydration, decarboxylation, and condensation reactions. Such chemical transformations contribute to the development of disordered yet stable carbon matrices and increase graphitic character. Additionally, sodium ions introduced during premodification may catalyze structural rearrangements, enhancing thermal inertia and carbon ordering.^62,63^

### 3.4 X-ray Diffraction (XRD)

The XRD patterns of the materials are presented in Figure 4. The biochars BC(400) and BC(215–400) (Figure 4a and b) displayed similar diffraction profiles, with peaks at 28.2°, 40.4°, 50.1°, and 66.3°, assigned to sylvite (KCl, COD 9003112), and at 29.3°, 39.2°, 43.1°, and 47.3°, corresponding to calcite (CaCO_3_, COD 1010962).^64,65^

**Figure 4:**
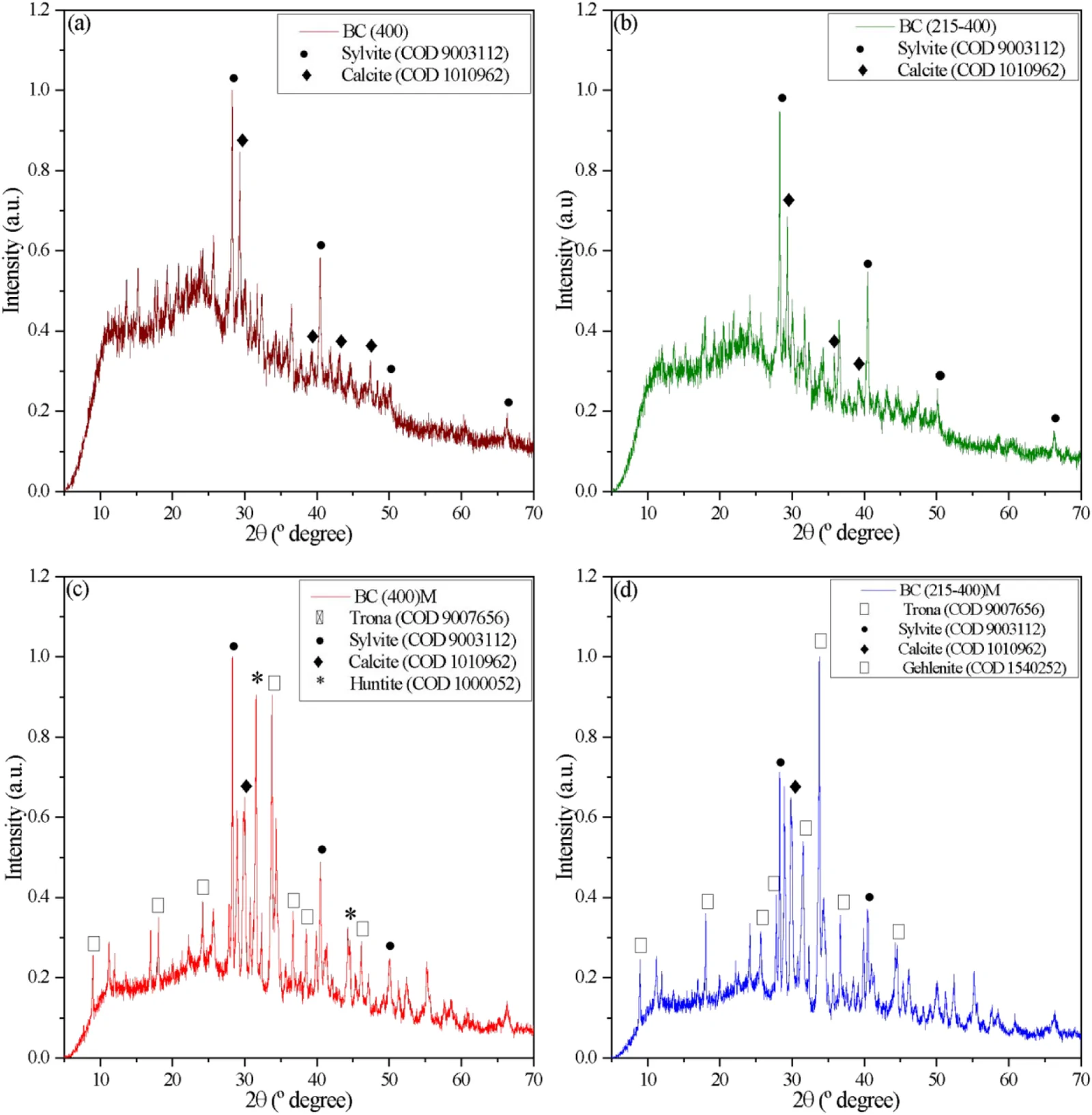
XRD patterns of the biochars (a) BC(400), (b) BC(215–400), (c) BC(400)­M and (d) BC(215–400)­M.

Upon modification with NaOH, the XRD patterns of BC(400)­M and BC(215–400)­M (Figure 4c and d) revealed additional peaks corresponding to trona (Na_3_H­(CO_3_)_2_·2H_2_O, COD 9007656) and huntite (Mg_3_Ca­(CO_3_)_4_, COD 1000052). Trona formation is likely due to the reaction of molten NaOH (at 400 °C) with CO_2_ released during pyrolysis or present in the furnace atmosphere, resulting in the hydrated carbonate upon cooling.^66,67^ Huntite formation can be explained by the reaction of magnesium and calcium ions naturally present in the biomass with CO_2_ under the strongly alkaline conditions provided by NaOH, promoting cation mobility and crystallization of mixed carbonates.^68^ These results indicate that NaOH treatment not only introduces sodium-based phases but also favors the formation of calcium–magnesium carbonates in the biochar.

### 3.5 Surface Area and Porosity Analysis

Table 3 summarizes the textural properties of the biochars produced under different pyrolysis conditions, including specific surface area, micro- and mesopore volumes, and pore diameter.

**Table 3:** Textural Properties of Biochars Produced under Different Pyrolysis Conditions, with and without NaOH Pretreatment

Sample	Specific surface area (m^2^ g^–1^)	V_micro_ (cm^3^ g^–1^)	V_meso_ (cm^3^ g^–1^)	Average pore diameter (nm)
BC(400)	1.51	0.00224	0.00524	18.13
BC(215–400)	2.84	0.00092	0.00823	3.41
BC(400) M	3.31	0.00198	0.03551	18.43
BC(215–400) M	5.43	0.00271	0.04566	17.51

The specific surface area (SSA) of the biochars ranged from 1.51 to 5.43 m^2^ g^–1^. The N_2_ adsorption–desorption isotherms (Figure S7a–d) exhibited Type IV behavior according to the IUPAC classification, indicating predominantly mesoporous structures. In addition, the hysteresis loops were characteristic of the H3 type, suggesting the presence of slit-shaped pores and aggregates of plate-like particles. The sharp increase in adsorbed volume at high relative pressure (P/P_0_ > 0.8) is associated with capillary condensation in mesopores, while the low micropore volumes obtained by the t-plot method indicate limited microporosity in all samples.^69^

Chemical modification with NaOH and use of stepwise pyrolysis led to a increase in SSA; particularly for the BC(215–400)­M sample, the surface area increased by approximately 3.6 times compared with BC(400). This enhancement can be attributed to the removal of volatile fractions and the unblocking of previously inaccessible pores, resulting in a more developed porous structure.^70,71^

The mesopore volume, determined by the Barrett–Joyner–Halenda (BJH) method, also increased in the modified samples. BC(215–400)­M reached 0.0457 cm^3^ g^–1^, compared to 0.0082 cm^3^ g^–1^ in its unmodified form. Meanwhile, the micropore volumes, estimated via the t-plot method, remained low in all samples (<0.003 cm^3^ g^–1^), indicating the predominance of mesoporosity even after modification.

Notably, BC­(400) presented a slightly higher micropore volume (0.0022 cm^3^ g^–1^), which, combined with its larger average pore diameter (18.13 nm), suggests a more heterogeneous pore distribution. The average pore diameter was mostly within the mesoporous range (17–18 nm), except for BC(215–400), which exhibited a significantly smaller value (3.41 nm), within the lower mesopore-size range.

The prevalence of mesoporosity in the modified samples is particularly advantageous for the adsorption of larger molecules such as TC, facilitating intraparticle diffusion and reducing mass transfer limitations.^72^

Although the SSA values are lower than those typically reported for conventional biochars (1–50 m^2^ g^–1^ for unmodified biochars and up to 260 m^2^ g^–1^ for NaOH-modified biochars), the adsorption performance obtained in this study demonstrates that high specific surface area is not the only factor governing TC adsorption.^73,74^ Overall, alkaline treatment with NaOH altered the porous structure of the materials, especially by enhancing mesoporosity, which may contribute to greater adsorption efficiency for antibiotics in aqueous environments.^75^

### 3.6 Determination of Surface Functional Groups and pH at the Point of Zero Charge (pH_PZC_)

The surface chemistry of the biochars was evaluated through pH_PZC_ determination (Figure S9) and conductometric titration of acidic and basic functional groups (Table S2 and Figure S10a–e).

Pyrolysis increased the pH_PZC_ relative to the in natura material (pH_PZC_ = 7.1), with values of 10.0 and 9.9 obtained for BC(400) and BC(215–400), respectively. These unmodified samples exhibited similar densities of acidic (2.6 and 2.4 mmol g^–1^, respectively) and basic groups (2.9 mmol g^–1^ for both), indicating limited variation in surface functionality due to the thermal treatment alone.^76,77^ NaOH-modified biochars exhibited higher pH_PZC_ values (10.4 and 11.7 for BC(400)­M and BC(215–400)­M, respectively) and greater concentration of basic surface groups (6.8 mmol g^–1^ for both modified materials), compared with the nonmodified counterparts. The concentration of acidic groups varied among modified samples, with values of 2.4 and 1.7 mmol g^–1^ for BC(400)­M and BC(215–400)­M, respectively. Consequently, the total concentration of basic and acidic surface functional groups reached 9.2 and 8.5 mmol g^–1^ for these materials, respectively.

This behavior suggests that alkaline pretreatment promotes both the incorporation of basic functionalities and the transformation or partial degradation of thermolabile acidic groups, depending on the thermal regime.^76^ Consequently, the shift toward a more alkaline surface character is attributed to the formation of oxygen-containing basic functional groups, such as phenolate and carboxylate species, induced by the alkaline pretreatment and subsequent slow pyrolysis process, in agreement with FTIR results.^78,79^

### 3.7 Initial Adsorption Capacity

A preliminary adsorption test was conducted to identify the biochars with the highest adsorption capacity for TC and to select materials for further investigation. The experiments were performed without previous pH adjustment. Due to the high pH_PZC_, especially for NaOH-modified biochars, the final pH increased significantly, reaching 10.5.^80,81^ This pH shift strongly affected TC speciation. As an amphoteric compound (p*K*_a_ values of 3.3, 7.7, and 9.7), TC predominantly exists as the doubly deprotonated anion (TC^2–^) at pH > 9.7, altering its structure, solubility, and interaction with the adsorbent surface.^82,83^ To ensure accurate quantification, the UV–vis analytical curve was prepared at pH 10.5, consistent with the final conditions of the adsorption systems.

At C_0_ = 100 mg L^–1^, NaOH-modified biochars exhibited significantly higher removal efficiencies (Figure S10), with BC(215–400)­M achieving 69% and BC(400)­M 35%, whereas the unmodified samples showed limited performance (6.6% for BC(400) and 0.9% for BC(215–400)). Since both materials were modified with NaOH, the better performance of BC(215–400)­M indicates a combined effect of alkaline activation and the two-step pyrolysis protocol, which promoted distinct physicochemical changes compared with direct pyrolysis at 400 °C.^20,84^ Among these properties, BC(215–400)­M exhibited a higher surface area and mesopore volume (Table 3), which may have increased the number of sites available for adsorption. Then, BC(400)­M and BC(215–400)­M were considered for the next experiments.

Because substantial pH drift occurred during adsorption in the unbuffered systems (Figure S12 and Tables S3 and S4), preventing comparison between the materials under equivalent pH conditions, buffered media were used for the pH-dependence, kinetic, and equilibrium experiments. The pH-dependence experiments were conducted using 0.01 mol L^–1^ B–R buffer adjusted to each target pH, whereas the kinetic and equilibrium experiments were performed in the same buffer at an initial pH of 6.0. The buffer limited pH variation, thereby providing better control over TC speciation and biochar surface charge. Since adsorption can be decreased in the buffered systems, possibly because of competition or other interactions involving the buffer components,^85–87^ a relatively low B–R buffer concentration was selected to balance pH control against potential matrix effects.

### 3.8 Effect of pH on Adsorption

The adsorption of TC by the NaOH-modified biochars was strongly governed by solution pH (Figure 5a–b), reflecting the coupled effects of adsorbent surface chemistry and TC speciation in aqueous solution. TC is an amphoteric antibiotic that undergoes successive acid–base equilibria and exists predominantly in cationic form below pH 3.3, as a zwitterion between pH 3.3 and 7.7, and increasingly in anionic forms under alkaline conditions.^88,89^ In parallel, the biochars exhibited alkaline pH_PZC_ values (>10.0), indicating that their surfaces remained predominantly positively charged across most of the tested pH range.

**Figure 5:**
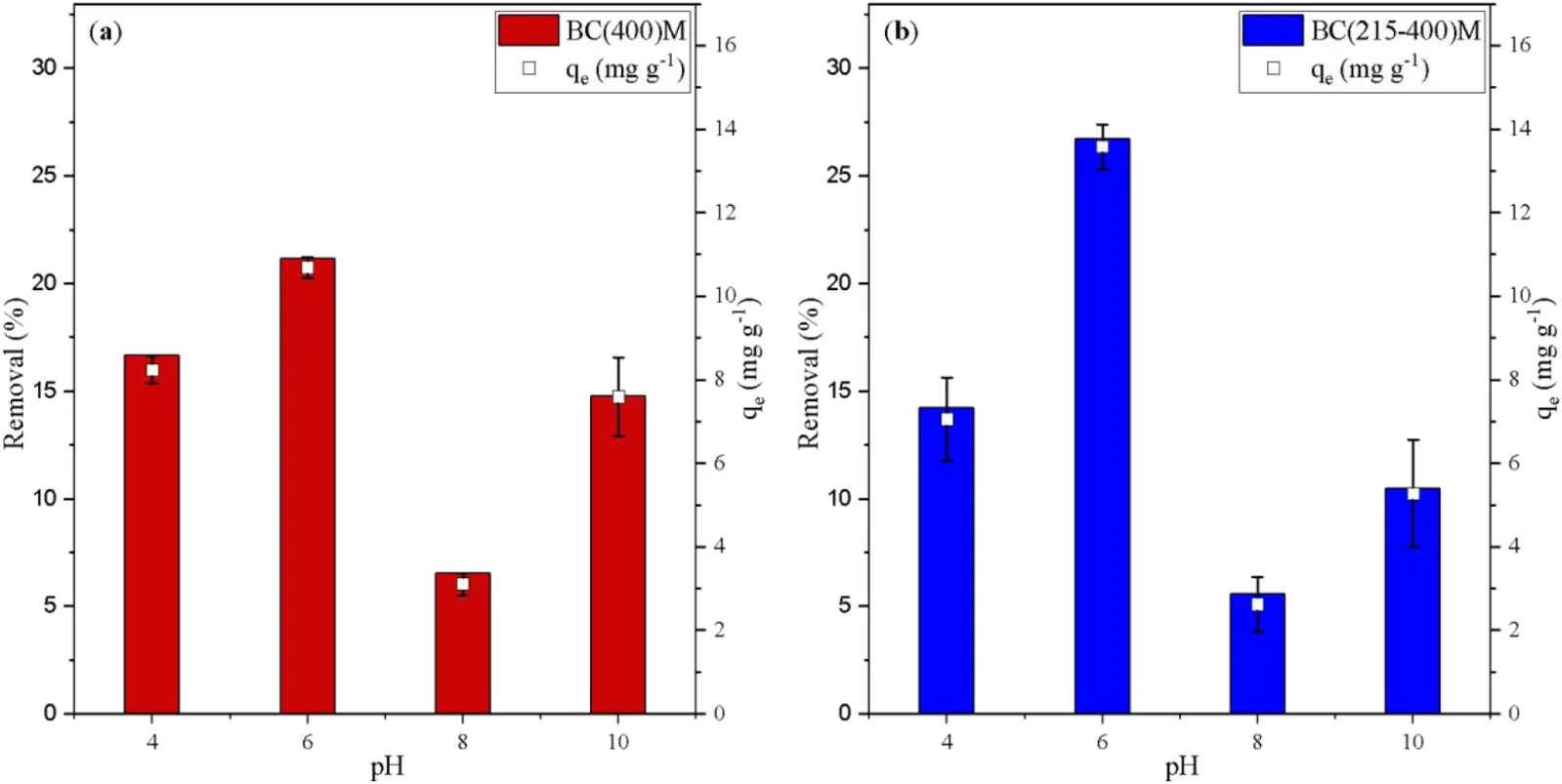
Effect of initial pH on TC adsorption for (a) BC(400)­M and (b) BC(215–400)­M. Conditions: initial TC concentration = 50 mg L^–1^; adsorbent dose = 1.0 g L^–1^; temperature = 25 °C; agitation speed = 120 rpm; contact time = 24 h. Error bars represent the standard deviation of three independent experiments (*n* = 3).

The final solution pH was measured after adsorption at each initial pH value for both biochars (Table S4). The 0.01 mol L^–1^ B–R buffer substantially attenuated, but did not eliminate, pH drift during contact with the biochars. For BC(400)­M, final pH values of 4.82 ± 0.02, 6.70 ± 0.06, 8.56 ± 0.02, and 10.34 ± 0.02 were obtained at initial values of 4.08, 6.00, 8.04, and 10.06, respectively. The corresponding final values for BC(215–400)­M were 4.78 ± 0.01, 6.85 ± 0.01, 8.58 ± 0.01, and 10.43 ± 0.02. Although deviations from the initial pH values were not negligible, the final pH values of the two biochars differed by no more than 0.15 pH units under each condition, enabling their comparison under closely matched solution conditions. Moreover, the pH shifts did not change the predominant TC speciation regime associated with each tested condition. For example, at an initial pH of 6.00, the final values of 6.70 and 6.85 remained below the second *pK_a_* of TC (approximately 7.7), and the zwitterionic form therefore remained predominant. Thus, buffering improved control over TC speciation and biochar surface charge, although the remaining pH shifts were considered when interpreting the adsorption results.

The highest adsorption capacities were observed at initial pH of 6.0, reaching 10.7 mg g^–1^ for BC(400)­M and 13.61 mg g^–1^ for BC(215–400)­M. Under these conditions, adsorption may involve site-specific electrostatic interactions between the localized charged groups of TC and surface sites, together with hydrogen bonding and π–π interactions between the aromatic domains of the biochars and the conjugated structure of TC.^90^ The superior adsorption of TC by BC(215–400)­M at an initial pH of 6.0 may result from a favorable balance between TC speciation and the surface charge of the biochar, combined with its larger surface area and mesopore volume, which enhance the accessibility of adsorption sites and facilitate hydrogen-bonding and π–π interactions.^91,92^

As the initial pH increased beyond 6.0, adsorption decreased, likely due to the progressive deprotonation of TC and the consequent weakening of favorable electrostatic interactions. At an initial pH of 8.0, adsorption decreased for BC(400)­M and BC(215–400)­M, consistent with the increasing predominance of anionic TC species and the reduced contribution of charge-driven interactions.^20,89^ The persistence of adsorption under these conditions can sustain adsorption through hydrogen bonding and π–π interactions, which are less dependent on electrostatic conditions.^93,94^

At an initial pH of 10.0, the higher adsorption capacity relative to that observed at initial pH of 8.0, despite the predominance of anionic TC species, may reflect changes in TC speciation and in the ionization, accessibility, and chemical environment of the surface sites. However, the available results do not allow this increase to be attributed to a specific interaction. Overall, the pH-dependent behavior suggests that TC adsorption is governed by the combined effects of TC speciation, surface charge and functionality, aromatic domains, and pore accessibility rather than by electrostatic interactions or surface area alone.

Finally, although BC(215–400)­M had a larger surface area and mesopore volume (Table 3), it did not consistently exhibit higher adsorption across the evaluated pH range. BC(400)­M performed better at initial pH of 4.0, 8.0, and 10.0, which may be related to its higher density of acidic and total surface functional groups (Section 3.6), whereas the more developed porous structure of BC(215–400)­M may have contributed to its higher adsorption at initial pH of 6.0.

Based on these considerations, an initial pH of 6.0 was selected for the kinetic and equilibrium experiments. Because the final pH was not measured at each kinetic time or initial TC concentration, time- and concentration-dependent pH variations beyond those observed in the representative end point experiments at initial pH of 6.0 cannot be excluded.

### 3.9 Adsorption Kinetics and Isotherms

The kinetic data for TC removal by the materials BC(400)­M and BC(215–400)­M are presented in Figure 6. The experimental data were fitted to the nonlinear pseudo-first order, pseudo-second order and intraparticle diffusion models, and the kinetic parameters and quality indicators (adjusted coefficient of determination, R^2^_adj_, and sum of squared errors, SSE) obtained are shown in Table 4.

**Figure 6:**
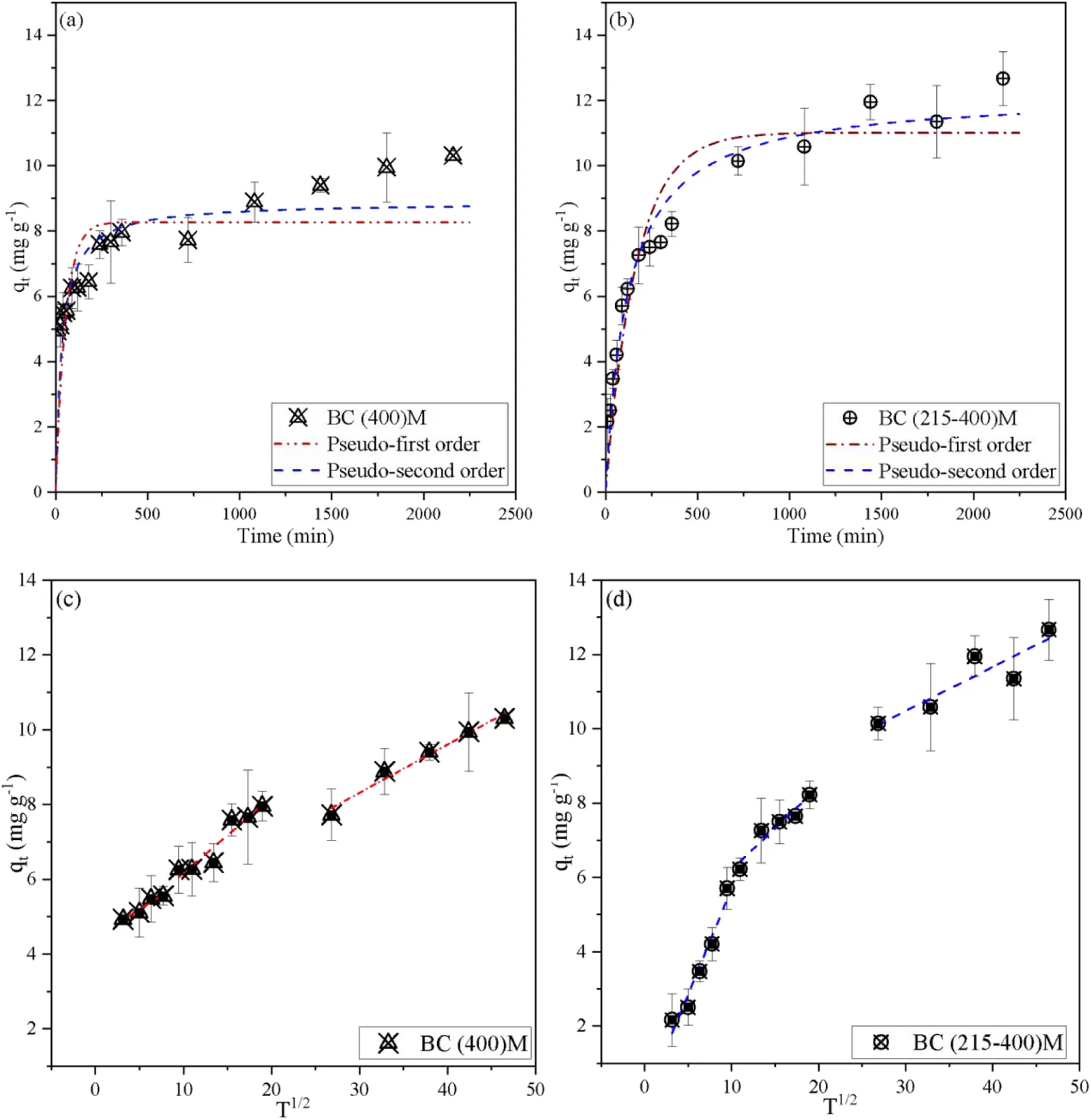
Adsorption kinetics of TC on biochars (a–b) and intraparticle diffusion plots (c–d). Conditions: initial TC concentration = 50 mg L^–1^; initial pH = 6.0; adsorbent dose =1.0 g L^–1^; temperature = 25 °C; agitation speed = 120 rpm. Error bars represent the standard deviation of three independent experiments (*n* = 3).

**Table 4:** Kinetic Parameters and Intraparticle Diffusion Model Constants for TC Adsorption on Biochars^a^

	Biochar	
Parameter	BC(400)M	BC(215–400)M
*q_e_* (*exp*) (mg g^–1^)	10.30	12.67
Pseudo-first order		
*q_e_* (mg g^–1^)	8.10	11.01
*k*_1_ (min^–1^)	0.0312	0.0060
R^2^_adj_	0.4583	0.9287
SSE (mg^2^ g^–2^)	28.26	16.79
Pseudo-second order		
*q_e_* (mg g^–1^)	8.74	12.21
*k*_2_ (g mg^–1^ min^–1^)	0.0050	0.0007
R^2^_adj_	0.6728	0.9675
SSE (mg^2^ g^–2^)	15.63	6.44
Intraparticle Diffusion		
*C*_1_ (mg g^–1^)	4.4498	0.0057
*k_dif_*_.1_ (mg g^–1^ min^–0.5^)	0.1287	0.5684
R^2^_adj_	0.9882	0.9749
*C*_2_ (mg g^–1^)	4.1284	3.9749
*k_dif_*_.2_ (mg g^–1^ min^–0.5^)	0.2032	0.2229
R^2^_adj_	0.9486	0.9613

a Nonlinear fittings were performed.

The PSO model provided a better fit for BC(215–400)­M, as indicated by higher adjusted R^2^_adj_ values and lower SSE compared to the pseudo-first-order model. However, for BC(400)­M, both models showed poor fitting (R^2^_adj_ < 0.673), indicating that simplified kinetic models are insufficient to fully describe the adsorption behavior. Although PSO is often associated with chemisorption, its applicability here more likely reflects the combined influence of surface reactivity and site availability rather than a single rate-limiting mechanism.^95^

The intraparticle diffusion model revealed two distinct linear regions for both materials, confirming a multistep adsorption process. The initial stage is governed by external mass transfer and faster surface adsorption, while the second stage corresponds to diffusion within the pore structure. BC(215–400)­M exhibited faster kinetics than BC(400)­M, reaching equilibrium within approximately 12 h, with a higher initial diffusion rate (*k_d_*_*if*.1_ = 0.5684 mg g^–1^ min^–0.5^) and a near-zero intercept (*C*_1_ = 0.0057 mg g^–1^), indicating negligible boundary layer resistance and efficient mass transfer.^96,97^

This behavior is consistent with FTIR and XRD results, which revealed the presence of oxygenated groups and carbonate phases (trona and calcite). These features increase surface polarity and can provide readily accessible adsorption sites, facilitating faster initial uptake through electrostatic and cation-mediated interactions.^20^ In contrast, BC(400)­M exhibited a significantly higher intercept (*C*_1_ = 4.45 mg g^–1^), indicating stronger boundary layer resistance and slower external mass transfer, resulting in a longer equilibrium time (18 h).

The adsorption isotherms for the biochars are shown in Figure 7.

**Figure 7:**
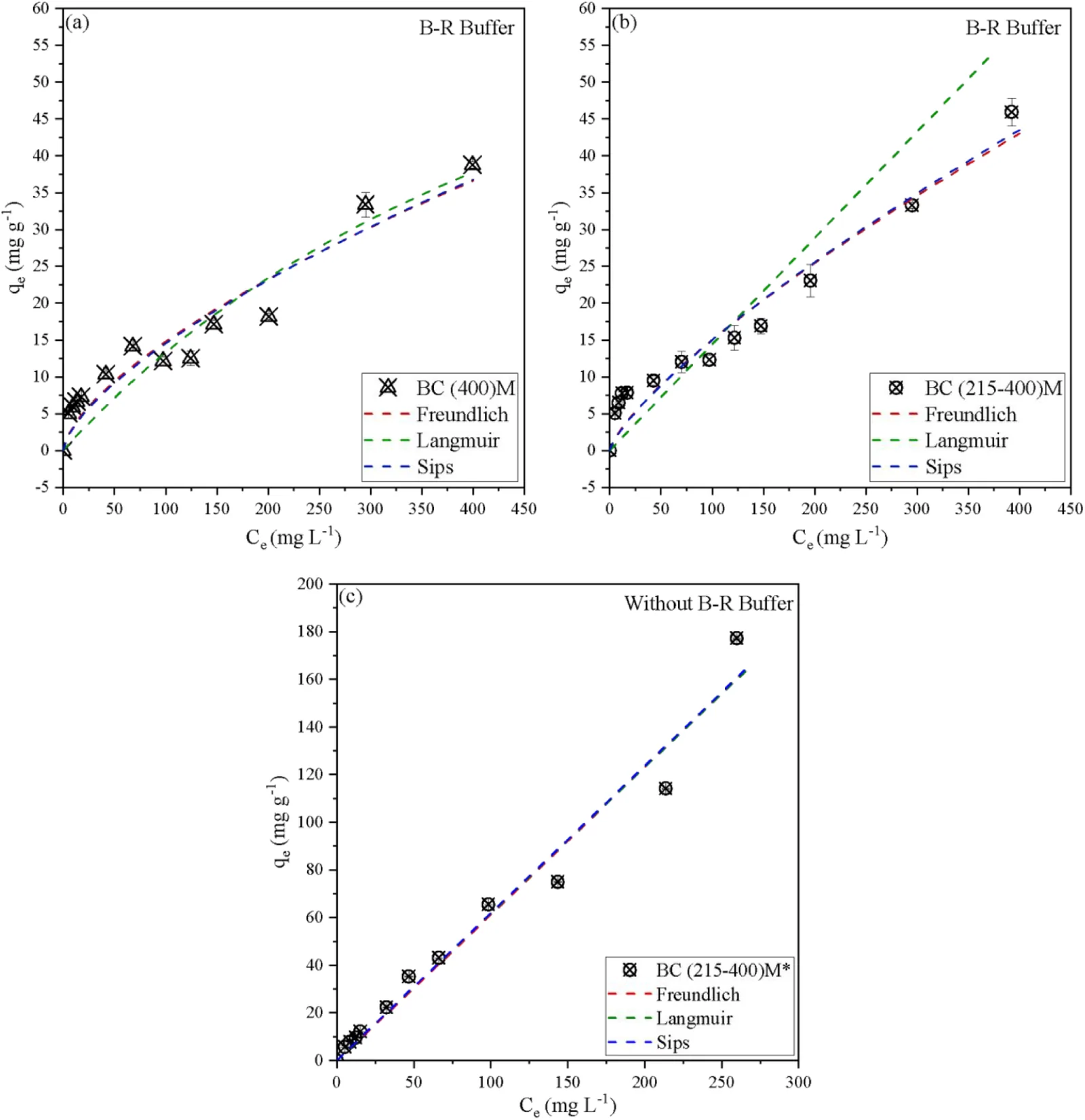
Adsorption isotherms of TC on biochars (a) BC(400)­M, (b) BC(215–400)­M and (c) BC(215–400)­M*. Conditions: initial TC concentration = 10–400 mg L^–1^; initial pH = 6.0; adsorbent dose = 1.0 g L^–1^; temperature = 25 °C; agitation speed = 120 rpm for 36 h. BC(215–400)­M* corresponds to unbuffered experimental analysis. Error bars represent the standard deviation of three independent experiments (*n* = 3).

The buffered isotherms (Figure 7a and b) were used to compare the intrinsic adsorption of the biochars under better-controlled pH conditions, whereas the unbuffered system (Figure 7c) was employed to assess adsorption in the absence of the buffering effect, with an initial pH of 6.0. According to the Giles classification, the buffered systems exhibited L-type isotherms, indicating favorable TC-biochar interactions.^98^

The unbuffered BC(215–400)­M* system displayed an approximately linear isotherm, consistent with a type C behavior, and reached a much higher experimental adsorption capacity (177.1 mg g^–1^) than the buffered systems (38.7 and 45.9 mg g^–1^ for BC(400)­M and BC(215–400)­M, respectively). This difference indicates that pH buffering reduced the apparent adsorption capacity by fixing the solution chemistry, whereas the unbuffered assay reflected a better operational uptake of the material. It should be emphasized that the value of 177.1 mg g^–1^ represents an apparent adsorption capacity. This value reflects the overall performance of the unwashed material under the specified experimental conditions and may include contributions from biochar-induced pH changes and residual soluble inorganic species; therefore, it should not be interpreted as an intrinsic property of the biochar.

The calculated data for the isotherm models are presented in Table 5.

**Table 5:** Parameters Obtained from the Fittings to the Freundlich, Langmuir and Sips Models^a^

		Biochar		
Model	Parameter	BC(400)M	BC(215–400)M	BC(215–400)M*
	*q_e_* (*exp*) (mg g^–1^)	38.7	45.9	177.1
Freundlich	*K_F_* [(mg g^–1^) (L mg^–1^)^1/n^]	0.725	0.454	0.57
	n	1.53	1.32	0.99
	R^2^_adj_	0.930	0.955	0.973
	SSE (mg^2^ g^–2^)	112.8	108.35	879.04
Langmuir	*q_max_* (mg g^–1^)	97.7	201.5	428.9
	*K_L_* (L mg^–1^)	1.57 × 10^–3^	7.12 × 10^–4^	6.4 × 10^–6^
	R^2^_adj_	0.926	0.962	0.973
	SSE (mg^2^ g^–2^)	170.7	146.3	916.4
Sips	*q_max_* (mg g^–1^)	629.4	3.28 × 10^3^	417.8
	*K_S_* (L mg^–1^)	2.34 × 10^–6^	9.06 × 10^–6^	2.3 × 10^–5^
	*n_S_*	0.673	0.77	0.99
	R^2^_adj_	0.930	0.955	0.971
	SSE (mg^2^ g^–2^)	117.2	110.8	917.4

a Nonlinear fittings were performed. BC(215–400)­M* = unbuffered experimental analysis.

The adsorption isotherms were analyzed using Freundlich, Langmuir, and Sips models, and the fitting quality was evaluated based on both R^2^_adj_ and SSE values. For BC(400)­M and BC(215–400)­M, all three models provided comparable fits, with very similar R^2^_adj_ values. However, the Freundlich model yielded the lowest SSE for both materials (112.8 and 108.35 mg^2^ g^–2^, respectively), indicating a slightly better representation of the experimental data. This suggests that adsorption in these materials is governed by surface heterogeneity, with multiple types of active sites contributing to the process rather than a uniform monolayer coverage.^99,100^

In contrast, the unbuffered system (BC(215–400)­M*) exhibited similar R^2^_adj_ values for all models but the Freundlich model provided the best fit (lowest SSE), and the *n* = 0.99 suggests a nearly linear adsorption behavior. This is consistent with a partition-like mechanism, in which adsorption sites are not saturated within the studied concentration range, in agreement with the Giles type C isotherm.^101^

### 3.10 Effect of Electrolyte Type on TC Adsorption

The influence of electrolyte type on TC adsorption was evaluated using CaCl_2_, MgSO_4_ and KH_2_PO_4_ solutions at initial pH of 6.0 (Figure 8).

**Figure 8:**
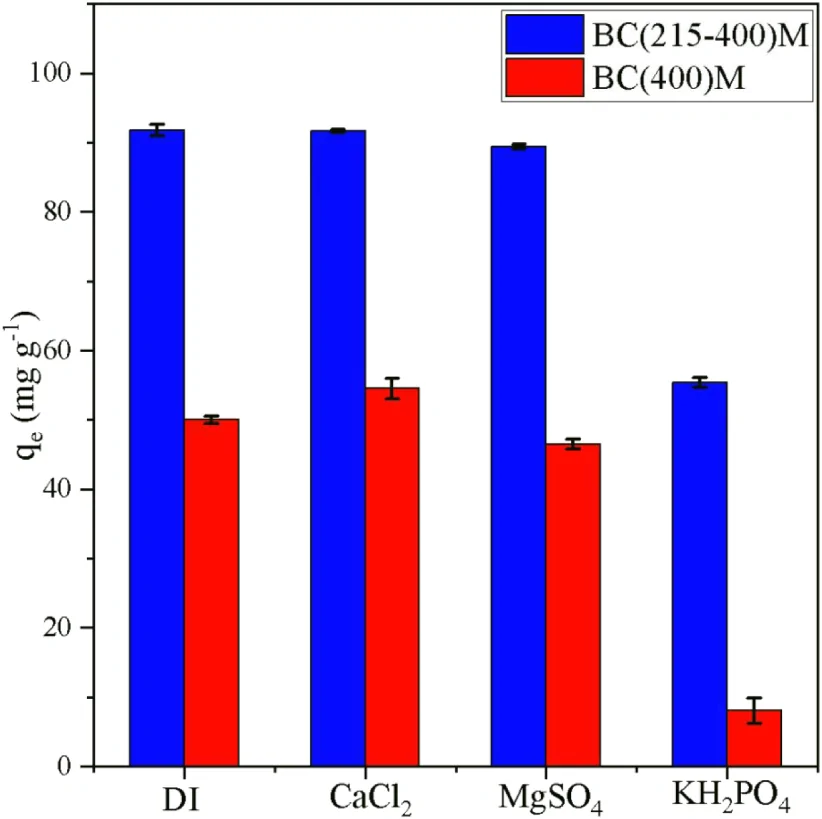
Effect of electrolyte type on TC adsorption onto BC(400)­M and BC(215–400)­M. Conditions: initial TC concentration = 100 mg L^–1^; initial pH = 6.0; adsorbent dose = 1.0 g L^–1^; temperature = 25 °C; and agitation speed = 120 rpm for 24 h. Error bars represent the standard deviation of three independent experiments (*n* = 3). DI refers to deionized water with no added electrolyte.

The adsorption behavior depended primarily on the identity of the electrolyte. For BC(215–400)­M, adsorption capacities of 54.0, 91.6, and 89.0 mg g^–1^ were obtained in the presence of KH_2_PO_4_, CaCl_2_, and MgSO_4_, respectively. BC(400)­M exhibited the same overall trend, with corresponding adsorption capacities of 7.9, 55.4, and 45.3 mg g^–1^. Compared with the electrolyte-free systems, in which adsorption capacities of 91.8 and 49.9 mg g^–1^ were obtained for BC(215–400)­M and BC(400)­M, respectively, CaCl_2_ and MgSO_4_ did not produce a consistent change in TC adsorption. In contrast, KH_2_PO_4_ caused a pronounced decrease in TC removal for both biochars.

At the initial pH of 6.0, TC predominantly occurs in its zwitterionic form. Nevertheless, because these experiments were unbuffered, the increase in pH during contact with the biochars must be considered (around 10.5; see Table S4) when interpreting TC speciation and adsorption behavior. Moreover, the electrolytes were evaluated at the same molar concentration but did not provide the same ionic strength. Consequently, the observed differences cannot be interpreted as an ionic-strength effect alone.

The absence of a consistent increase in adsorption in the presence of CaCl_2_ and MgSO_4_ provides no direct evidence that Ca^2+^ or Mg^2+^ promoted TC adsorption through cation bridging. Although TC is known to coordinate divalent metal ions, the present experiments do not distinguish between complex formation in solution and possible surface-mediated interactions.^102^

In contrast, the lower adsorption observed in KH_2_PO_4_ solution suggests that phosphate species compete with TC for oxygen-containing surface functional groups, reducing the number of available adsorption sites. This finding is particularly relevant because the adsorption experiments discussed in the previous sections were performed using Britton–Robinson buffer, which contains phosphoric acid. Therefore, the lower adsorption capacities obtained under buffered conditions may be partially associated with the presence of phosphate species rather than with pH control alone.^103^

### 3.11 Adsorption Performance in Tap Water

To preliminarily assess the influence of a more complex aqueous matrix on adsorption, experiments were conducted using tap water with an initial pH of 7.29, electrical conductivity of 761 μS cm^–1^, and turbidity of 5 NTU. The pH and electrical conductivity remained unchanged after preparation of the 100 mg L^–1^ TC solution, whereas turbidity was not measured because of interference from the coloration of the solution. In this matrix, BC(215–400)­M and BC(400)­M achieved TC removal efficiencies of 57.6% (*q_e_* = 56.5 mg g^–1^) and 16.0% (*q_e_* = 15.7 mg g^–1^), respectively. Under otherwise comparable conditions in deionized water, the corresponding removal efficiencies were 69.0 and 35.0% (see Section 3.7).

The decrease observed in tap water was more pronounced for BC(400)­M than for BC(215–400)­M. This matrix effect may be associated with dissolved inorganic constituents competing with TC for adsorption sites or altering TC speciation, surface properties, and the chemical environment of the adsorption system.^104^ However, the present experiment does not distinguish among these possible contributions. BC(215–400)­M retained a larger proportion of its adsorption performance in tap water, indicating lower sensitivity to the matrix composition than BC(400)­M under the evaluated conditions. These findings identify BC(215–400)­M as the more promising material for further evaluation in complex aqueous matrices. Nevertheless, studies using environmentally relevant TC concentrations and actual wastewater samples are required to establish its practical applicability.

### 3.12 Leaching of Inorganic Species from the Biochars

Short-term leaching experiments conducted in deionized water and TC solution were used to evaluate the aqueous release of inorganic elements from BC(400)­M, BC(215–400)­M, and BW(215–400)­M under the adsorption conditions (Table 6). A pronounced difference in alkali-metal release was observed between the unwashed and washed materials. In deionized water, BC(215–400)­M released 146 ± 4 mg g^–1^ of Na and 76 ± 4 mg g^–1^ of K, whereas BW(215–400)­M released only 1.69 ± 0.07 and 1.51 ± 0.08 mg g^–1^, respectively. A similar difference was observed in the TC-containing systems, in which Na and K release decreased from 132 ± 2 and 68 ± 2 mg g^–1^ for BC(215–400)­M to 8 ± 4 and 1.8 ± 0.1 mg g^–1^ for BW(215–400)­M, respectively, corresponding to reductions of approximately 93.9% and 97.3%.

**Table 6:** Leaching of Inorganic Elements from BC(400)­M, BC(215–400)­M, and BW(215–400)­M into Deionized Water and a 50 mg L^–1^ TC Solution^a^

	BC(400)M		BC(215–400)M		BW(215–400)M	
Element	DI	TC	DI	TC	DI	TC
Na	148 ± 3	132 ± 5	146 ± 4	132 ± 2	1.69 ± 0.07	8 ± 4
K	36.16 ± 0.03	36.17 ± 0.02	76 ± 4	68 ± 2	1.51 ± 0.08	1.8 ± 0.1
Ca	BDL	BDL	BDL^b^	0.75 ± 0.08	BDL	1.53 ± 0.07
Mg	0.43 ± 0.05	0.39 ± 0.01	0.48 ± 0.08	0.61 ± 0.03	0.46 ± 0.01	0.73 ± 0.07

a Both media were unbuffered and adjusted to an initial pH of 6.0. Results are expressed as milligrams of element released per gram of biochar (mg g^–1^) and presented as mean ± standard deviation (*n* = 3).

b BDL: below detection limit.

BC­(400)­M exhibited Na release comparable to that of BC(215–400)­M, with values of 148 ± 3 and 132 ± 5 mg g^–1^ in deionized water and TC solution, respectively. By contrast, its K release was approximately 47–53% lower than that of BC(215–400)­M. Overall, washing markedly reduced the subsequent release of water-leachable Na and K from BC(215–400)­M. These results demonstrate that postpyrolysis washing effectively removed most of the readily soluble Na- and K-containing species.

The high Na release from BC(215–400)­M is consistent with the presence of trona [Na_3_H­(CO_3_)_2_·2H_2_O] identified by XRD, indicating that part of the sodium introduced during NaOH pretreatment remained in the biochar as a readily soluble mineral fraction. The release of K, despite the absence of K-containing reagents during modification, is likely associated with native mineral species present in the banana pseudostem and concentrated in the biochar during pyrolysis. The similarly high Na release observed for BC(400)­M indicates that substantial amounts of readily soluble sodium-containing species were retained in both unwashed biochars after NaOH modification. In contrast, the considerably higher K release from BC(215–400)­M compared with BC(400)­M suggests that the two-step heating pathway influenced the retention or redistribution of potassium-containing inorganic species.

Ca was below the detection limit for BC(400)­M under both conditions and for BC(215–400)­M and BW(215–400)­M in deionized water, but was detected in the TC-containing suspensions of the latter two materials. Similarly, Mg release increased in the presence of TC for both BC(215–400)­M and BW(215–400)­M, whereas BC(400)­M showed similar Mg release under both conditions. These results indicate that TC affected the net mobilization of divalent elements during contact with the biochars. Because TC can coordinate Ca^2+^ and Mg^2+^, the formation of soluble TC-metal complexes may have stabilized these ions in the aqueous phase, reducing their readsorption or precipitation and increasing their measured concentrations in the supernatant.^20,105^

### 3.13 Effect of Postpyrolysis Washing on TC Adsorption

To further investigate the influence of the residual inorganic fraction on TC adsorption, the NaOH-modified biochars were washed with deionized water before the adsorption experiments. Figure 9 compares the TC adsorption capacity of washed and unwashed NaOH-modified biochars under buffered and unbuffered conditions. Under unbuffered conditions, washing had little effect on the adsorption performance of BC(400)­M, whose adsorption capacity changed from 49.9 to 48.7 mg g^–1^. In contrast, washing BC(215–400)­M reduced its adsorption capacity from 91.8 to 81.7 mg g^–1^.

**Figure 9:**
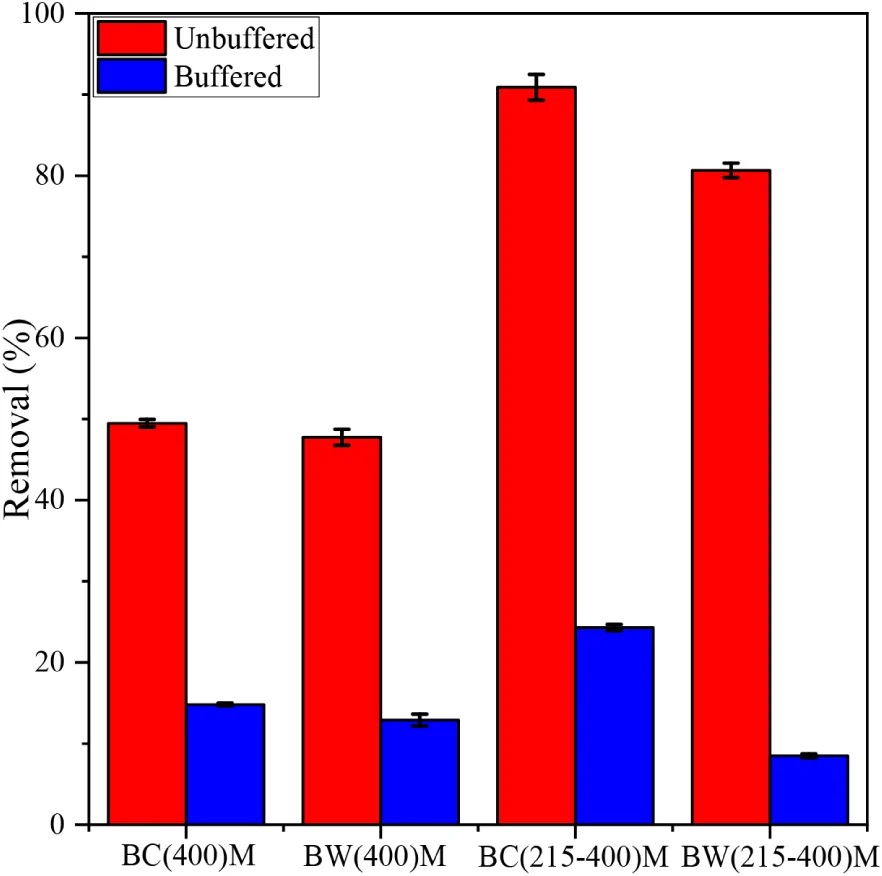
TC adsorption capacities of washed and unwashed NaOH-modified biochars under buffered and unbuffered conditions. Experimental conditions: initial TC concentration, 100 mg L^–1^; adsorbent dose, 1.0 g L^–1^; initial pH, 6.0; temperature, 25 °C; agitation rate, 120 rpm; and contact time, 24 h. Buffered experiments were conducted using 0.01 mol L^–1^ Britton–Robinson buffer. Error bars represent the standard deviations of three independent experiments (*n* = 3). The BW term denotes biochars washed with deionized water before adsorption.

Under buffered conditions, the effects of washing were more pronounced and strongly dependent on the pyrolysis pathway. Washing reduced the adsorption capacity of BC(400)­M from 14.7 to 12.6 mg g^–1^, corresponding to a 14.3% decrease. For BC(215–400)­M, however, the capacity decreased from 24.1 to 8.4 mg g^–1^, representing a 65.1% reduction. Consequently, although unwashed BC(215–400)­M outperformed BC(400)­M, this ranking was reversed after washing under buffered conditions. This result indicates that water-removable constituents made a substantial contribution to TC uptake by BC(215–400)­M in the presence of the B–R buffer.

The decrease in adsorption capacity after washing, particularly for BC(215–400)­M, cannot be interpreted as direct evidence that residual inorganic species acted as TC-binding sites. After washing, XRD reflections assigned to water-soluble crystalline phases were attenuated or no longer detected, whereas the principal FTIR bands associated with the carbonaceous matrix were retained (Figure S7; see the corresponding discussion in the Supporting Information). Nevertheless, washing may also have altered the ionic environment established during adsorption, reduced the abundance of surface-associated mineral phases, and changed the accessibility of carbonaceous adsorption sites. The removal of Ca- and Mg-containing species may have contributed to the decrease in adsorption capacity; however, this contribution was not directly demonstrated. Moreover, the leaching experiments indicated that TC affected the net mobilization of Ca and Mg from the material surface (Table 6) but did not establish their direct participation in surface complexation or cation-bridging mechanisms.

Under unbuffered conditions, BW(215–400)­M retained an adsorption capacity approximately 68% higher than that of BW(400)­M. However, the effect of washing must be interpreted together with the substantial changes in final pH. The final pH values of the unwashed BC(400)­M and BC(215–400)­M systems were 10.11 and 10.15, respectively, whereas washing decreased these values to 7.54 and 8.35 (Table S4). At the higher pH values reached by the unwashed biochars, TC is predominantly anionic and the biochar surfaces are expected to carry a greater negative charge, thereby increasing electrostatic repulsion. Consequently, the decrease in pH caused by washing would tend to favor TC adsorption and partially offset the capacity loss associated with the removal of water-removable constituents. The relatively modest 11% reduction observed for BC(215–400)­M under unbuffered conditions may therefore underestimate the influence of this fraction.

In the buffered systems, the final pH remained within a narrower range of 6.16–6.85, substantially reducing, although not completely eliminating, this compensatory pH effect. Under these conditions, washing decreased the adsorption capacity of BC(215–400)­M by approximately 65% and reversed the performance ranking of the two biochars. These findings indicate that the influence of washing results from coupled changes in pH, solution composition, and the abundance or accessibility of water-removable surface-associated species. Therefore, although the persistence of higher adsorption by BW(215–400)­M under unbuffered conditions supports the contribution of structural, chemical, and textural properties retained after washing, the specific contribution of residual inorganic species to TC uptake cannot be isolated from this experiment alone.^106^

From a practical perspective, the choice between washed and unwashed NaOH-modified biochars involves a trade-off between process simplicity, material stability, and adsorption performance. Using the biochars without postpyrolysis washing avoids additional water consumption and the generation of alkaline wastewater requiring further treatment and may preserve part of their adsorption performance. Moreover, for acidic TC-containing wastewaters, the alkaline character of the unwashed biochar could partially neutralize the influent, potentially reducing the need for prior pH adjustment. However, its use may also substantially increase the final pH and release soluble inorganic species, particularly Na and K, which could affect effluent quality and require downstream correction. Postpyrolysis washing markedly reduces this leaching and improves the chemical stability of the material, although it introduces an additional processing step and caused a modest decrease in adsorption capacity under the evaluated conditions. Therefore, the suitability of washing should be determined according to the composition and initial pH of the wastewater, effluent-quality requirements, water availability, and the need to minimize secondary waste generation.

### 3.14 Possible Adsorption Mechanism of TC on BC(215–400)­M

At an initial pH of 6.0, TC predominantly exists in its zwitterionic form. However, in the unbuffered system, the final pH increased to approximately 10.5 after contact with the biochar. Thus, anionic TC species were expected to predominate at equilibrium. Additionally, because this final pH remained below the pH_PZC_ of BC(215–400)­M (final pH = 11.7), the biochar was expected to retain an overall positive surface charge. This combination of TC speciation and surface charge is compatible with a contribution from electrostatic attraction, although this interaction was not independently isolated. Figure 10a compares the ATR-FTIR spectra of as-produced BC(215–400)­M, a TC-free process control, BC(215–400)­M after exposure to two initial TC concentrations, and pure TC used as a spectral reference.

**Figure 10:**
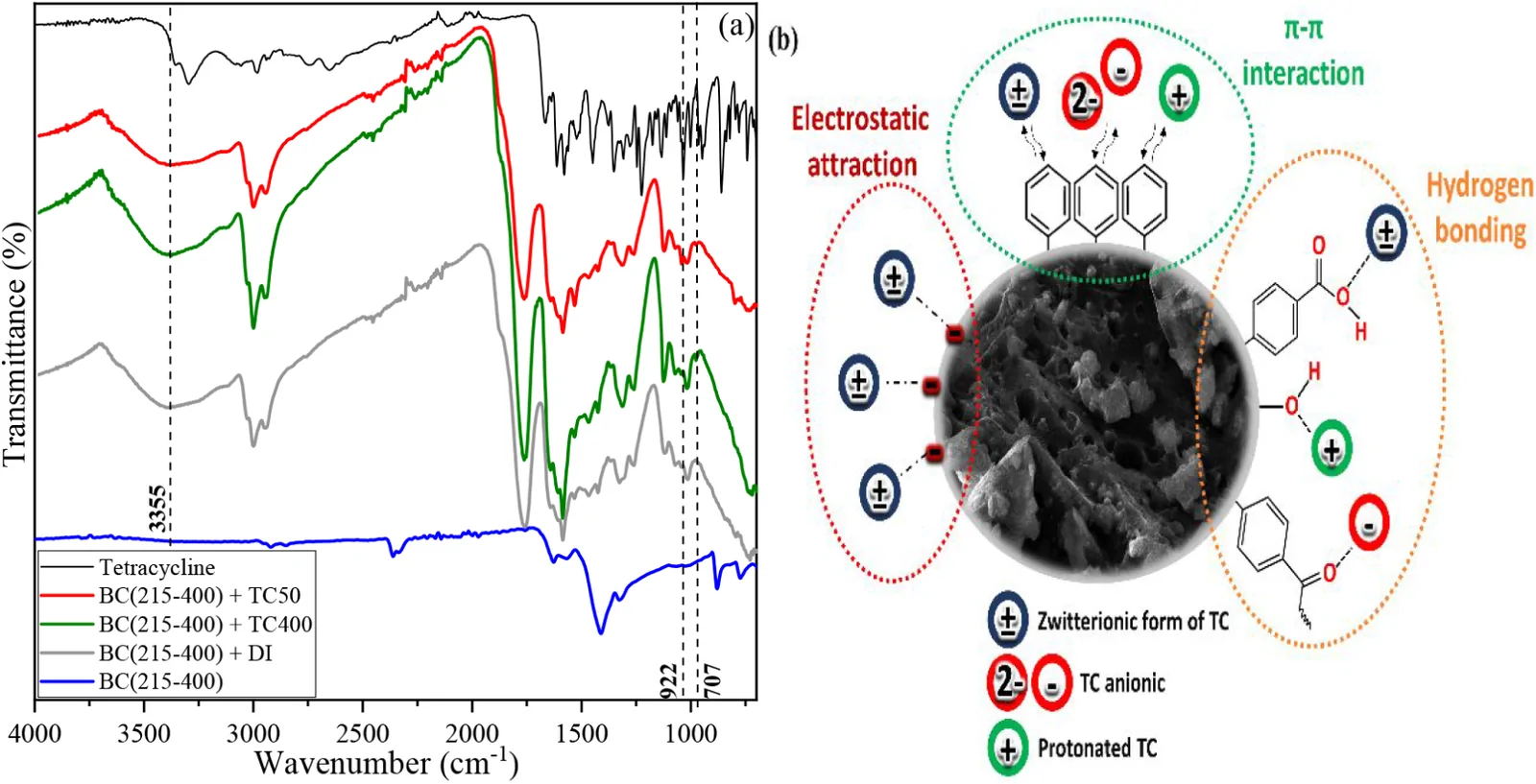
(a) ATR-FTIR spectra of as-produced BC(215–400)­M; BC(215–400)­M subjected to aqueous contact, recovery, washing, and drying in the absence of TC (BC(215–400)­M + DI); BC(215–400)­M after exposure to initial TC concentrations of 50 and 400 mg L^–1^ followed by the same recovery, washing, and drying procedures (BC(215–400)­M + TC50 and BC(215–400)­M + TC400, respectively); and pure TC as a spectral reference. (b) Conceptual representation of plausible contributions to TC adsorption by BC(215–400)­M. Experimental conditions: initial TC concentration, 50 or 400 mg L^–1^; initial pH, 6.0; adsorbent dose, 1.0 g L^–1^; temperature, 25 °C; agitation rate, 120 rpm; and contact time, 24 h. The TC-free control was prepared using deionized water adjusted to the same initial pH and was subjected to the same processing steps. No buffer was used.

The controlled comparison comprised as-produced BC(215–400)­M; BC(215–400)­M subjected to the same aqueous contact, recovery, washing, and drying procedures in the absence of TC (BC(215–400)­M + DI); and BC(215–400)­M exposed to initial TC concentrations of 50 and 400 mg L^–1^, followed by the same recovery, washing, and drying procedures (BC(215–400)­M + TC50 and BC(215–400)­M + TC400, respectively). This experimental design helped distinguish changes caused by aqueous processing and washing from those associated with TC exposure.

The principal spectral features of the biochar were preserved after aqueous contact and washing. However, differences between the TC-exposed samples and the TC-free process control were observed, particularly in the regions around 922 and 840 cm^–1^. A weak feature near 707 cm^–1^ was also observed in both TC-exposed samples but was not evident in the control. The occurrence of these differences after standardized washing is consistent with spectral changes associated with TC exposure. These changes may arise from retained TC-associated species or from TC-induced alterations in the vibrational environments of surface functionalities and inorganic constituents. Because these spectral regions contain potentially overlapping contributions from the carbonaceous matrix, mineral phases, and TC-associated species, FTIR alone cannot distinguish among these possibilities. Therefore, the results provide qualitative evidence of TC-associated changes persisting after washing but do not independently identify intact TC on the biochar or establish a specific adsorption mechanism.

To obtain additional evidence of TC retention, thermogravimetric analysis was performed on BC(215–400)­M after exposure to TC and on a TC-free process control subjected to the same contact, recovery, washing, and drying procedures (Figure S13). A mass-loss event centered at approximately 203 °C was observed in the DTG curve of the TC-exposed sample but was absent from the control. Because both samples underwent identical processing, this additional event is consistent with the retention of TC-associated material after adsorption and washing.^107^

Furthermore, elemental analysis provided complementary evidence of TC retention after washing. The TC-free BC(215–400)­M control contained 0.19% N (w/w), whereas the sample exposed to an initial TC concentration of 50 mg L^–1^ contained 0.36% N (w/w). Assuming that the nitrogen enrichment originated exclusively from TC-associated species retained after washing, the corresponding TC-equivalent amount was estimated from the increase in N content. An increase of 0.01% N corresponds to 0.10 mg N g^–1^ of sample. Therefore, the observed increase of 0.17 percentage points corresponds to 1.70 mg N g^–1^. Because each TC molecule contains two nitrogen atoms, this value was divided by 28.014 mg N mmol^–1^ TC, yielding an estimated retention of 0.061 mmol g^–1^, equivalent to approximately 29.3 mg g^–1^ when expressed as tetracycline hydrochloride.

This nitrogen-derived estimate was higher than the adsorption capacity of approximately 22 mg g^–1^ determined by the solution-depletion method. The discrepancy may reflect the uncertainty associated with calculating TC retention from a relatively small difference between low nitrogen contents, sample heterogeneity, and changes in the relative elemental composition caused by the release of inorganic constituents during contact and washing. Uncertainty in the aqueous TC determination and differences between the independently prepared samples may also have contributed. Moreover, the elemental calculation assumes that all additional nitrogen originated from retained TC and that the native nitrogen fraction remained unchanged. The value of 29.3 mg g^–1^ should therefore be regarded as a semiquantitative TC-equivalent estimate rather than an independent mass-balance measurement. Nevertheless, the increase in N content after TC exposure, despite identical washing of the control and TC-loaded samples, supports the persistence of TC-associated species in the recovered biochar.

The electrolyte experiments (Section 3.10) showed that the presence of CaCl_2_ or MgSO_4_ did not substantially alter the adsorption capacity, indicating that these electrolytes neither promoted nor inhibited TC removal under the evaluated conditions. In contrast, a marked decrease in adsorption capacity was observed in the presence of KH_2_PO_4_. This reduction may be associated with specific phosphate effects, including competition for adsorption sites, modification of surface charge, or interactions with inorganic components of the biochar. Because the evaluated electrolytes differed in both ionic composition and ionic strength, this behavior cannot be attributed to ionic strength alone. Moreover, the absence of enhanced adsorption in the presence of Ca^2+^ and Mg^2+^ provides no direct support for cation-bridging mechanisms. Although TC can coordinate divalent cations, the present experiments do not distinguish between soluble TC-metal complex formation and possible surface-mediated interactions, as presented in other works.^108–111^

Overall, the results indicate that TC adsorption onto BC(215–400)­M likely depends on the combined effects of TC speciation, biochar surface charge, oxygen-containing functionalities, aromatic domains, pore accessibility, and the composition of the aqueous medium. Figure 10b summarizes the plausible contributions to TC adsorption inferred from the combined adsorption and characterization results. The scheme is conceptual and does not establish the occurrence or relative importance of the individual interactions represented. Electrostatic attraction, hydrogen bonding, π–π interactions, and cation-mediated processes are therefore presented as plausible adsorption pathways rather than individually confirmed mechanisms. The adsorption capacity retained after postpyrolysis washing further indicates that the performance of BC(215–400)­M cannot be attributed solely to readily soluble inorganic species and is substantially associated with the structural, chemical, and textural properties retained after NaOH pretreatment and two-step pyrolysis.

### 3.15 Regeneration and Reusability of the Biochar

The reusability of the biochars was evaluated over three consecutive adsorption–desorption cycles (Figure S14). BC(215–400)­M exhibited TC removal efficiencies of 95.2% and 85.0% in the first and second cycles, respectively, whereas BC(400)­M showed lower but relatively stable removal efficiencies of 71.5% and 70.2%. In the third cycle, the performance of both materials decreased markedly, with removal efficiencies reaching 29.1% for BC(215–400)­M and 18.9% for BC(400)­M.

The higher removal achieved by BC(215–400)­M during the first two cycles is consistent with its greater adsorption performance before regeneration. However, the present results do not establish that this behavior resulted from greater structural stability. The loss of performance after repeated reuse may be associated with incomplete TC desorption and the consequent persistence of occupied or blocked adsorption sites.^112^ Repeated contact with ethanol and water may also modify surface functionalities or remove residual inorganic species, thereby altering the adsorption environment in subsequent cycles.^113,114^ Nevertheless, these possible changes were not directly characterized and should therefore be regarded as tentative explanations.

Overall, BC(215–400)­M retained a substantial removal efficiency during the first two cycles but underwent a pronounced decline in the third cycle. These findings indicate preliminary potential for short-term reuse; however, optimization of the desorption procedure and further evaluation over additional cycles are necessary before its practical applicability and long-term stability can be established.

### 3.16 Comparative Adsorption Performance

In this study, the maximum pyrolysis temperature was limited to 400 °C to reduce the thermal severity of biochar production. In addition, banana pseudostem is an abundant and underutilized agricultural residue, making it an attractive low-cost precursor. The comparison with other alkali-modified biochars (Table 7) shows that BC(215–400)­M achieved an adsorption capacity within the range reported for materials produced at higher temperatures. Nevertheless, direct comparisons based exclusively on maximum adsorption capacity should be interpreted cautiously because the studies differed in adsorbent dose, equilibrium time, initial TC concentration, pH-control conditions, and postproduction washing. Moreover, the value of 177.1 mg g^–1^ obtained for BC(215–400)­M represents the apparent capacity of the unwashed material under unbuffered conditions and should not be regarded as an intrinsic material property.

**Table 7:**
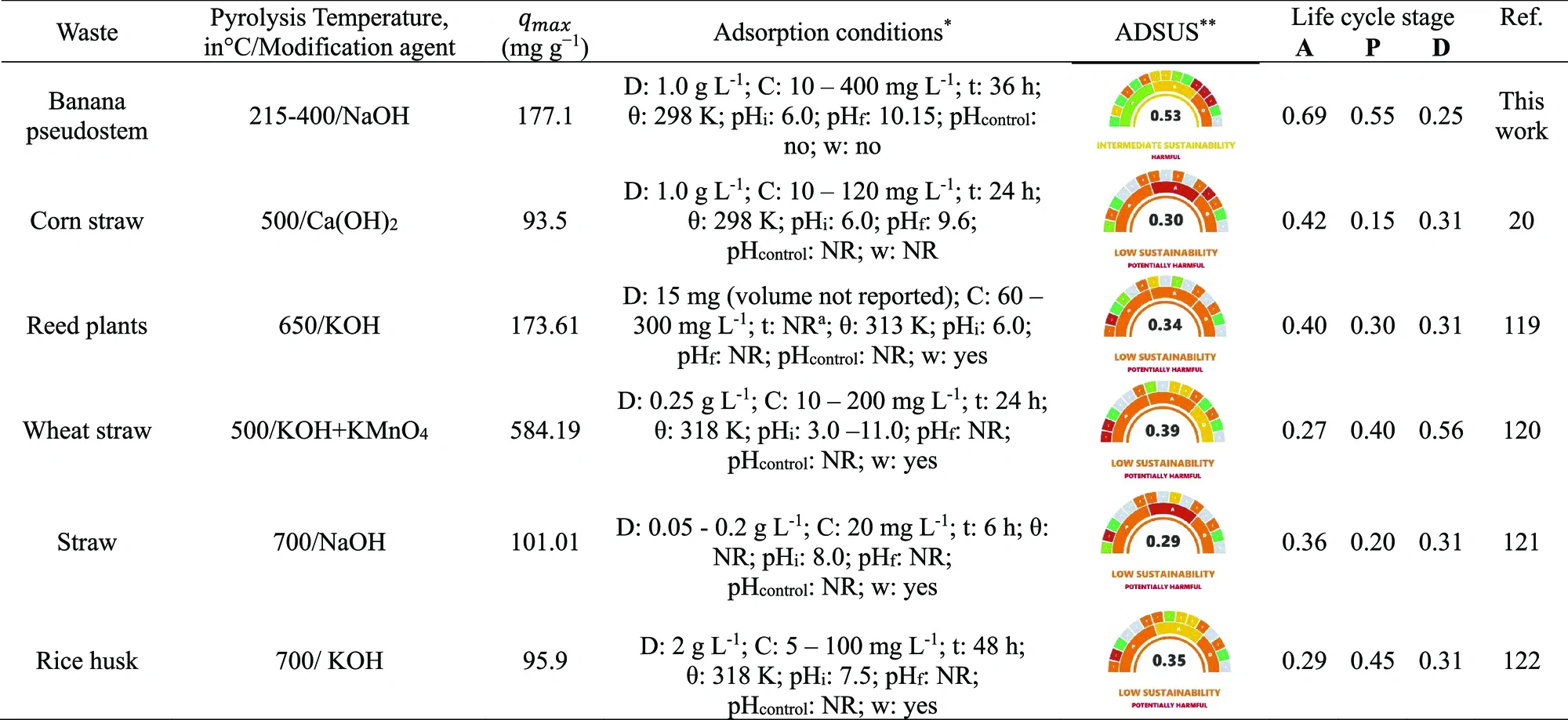
Comparative Analysis of the Maximum Adsorption Capacity of TC onto Alkali-Modified Biochars Derived from Waste Materials^a^ ^20,116–119^

a D: adsorbent dose; C: TC concentration or concentration range evaluated; t: equilibrium contact time used in the adsorption isotherm experiments; θ: temperature; pH_i_: optimum initial pH identified in separate experiments, but not explicitly reported as the pH used in the isotherm experiments; pH_f_: final pH measured in separate experiments; pH control: method used to control pH during the isotherm experiments; w: whether the adsorbent was washed before use. NR = Not reported^.^^**^The ADSUS values and graphical indicators were generated using the ADSUS software made available by the de Jesus et al.^115^

To provide a broader comparison, the materials were also evaluated using the Adsorbent Sustainability Index (ADSUS), a multicriteria framework that considers the production (P), application (A), and disposal (D) stages of the adsorbent life cycle.^115^ BC(215–400)­M achieved stage scores of 0.55 for production, 0.69 for application, and 0.25 for disposal. Among the biochars included in Table 7, it exhibited the highest application score (0.69 versus 0.27–0.42) and a comparatively favorable production score (0.55 versus 0.15–0.45). These results reflect the combination of an abundant waste precursor, a maximum pyrolysis temperature of 400 °C, the use of a single alkaline modifier, and the adsorption performance of the resulting material. By comparison, the KOH/KMnO_4_-modified wheat-straw biochar exhibited a substantially higher adsorption capacity, but its preparation involved a dual chemical treatment, a strong oxidizing agent, and a higher pyrolysis temperature, illustrating why adsorption capacity alone does not provide a comprehensive basis for evaluating an adsorbent. Conversely, the relatively low disposal-stage score of BC(215–400)­M (0.25, compared with 0.31–0.56 for the other materials) identifies end-of-life management as the main limitation of the proposed material.

It should be noted that several ADSUS criteria could not be evaluated for the literature-reported materials because the required experimental or process information was not provided in the original studies. These unavailable criteria are represented by gray boxes in the pictogram. Therefore, the absence of a score for these criteria should not be interpreted as poor performance, but rather as insufficient information for assessment. Consequently, comparisons among the materials should be regarded as indicative rather than definitive, because differences in reporting completeness may influence the resulting stage scores. This limitation also highlights the importance of reporting preparation conditions, water and chemical consumption, regeneration procedures, leaching behavior, and end-of-life management when assessing adsorbent sustainability.

Thus, the ADSUS assessment does not establish the absolute superiority of BC(215–400)­M but indicates a favorable balance between production requirements and application performance while highlighting disposal, long-term regeneration, and leaching control as priorities for further development.

### 3.17 Cost Analysis

A preliminary estimate of the direct input costs was performed to identify the main economic contributors to BC(215–400)­M production. Banana pseudostem was considered a locally abundant, low-value agricultural residue, and a zero feedstock purchase price was assumed. This assumption does not account for collection, transportation, storage, cleaning, or size-reduction costs. Based on the alkaline pretreatment procedure, approximately 100 g of NaOH was used per kilogram of dry biomass. Considering a biochar yield of 68%, this corresponds to approximately 147 g of NaOH per kilogram of as-produced biochar and an estimated cost of USD 0.12–0.18 kg^–1^. Pretreatment required approximately 12.5 L of water per kilogram of biomass, equivalent to 18.4 L kg^–1^ of biochar, with an estimated cost of USD 0.04–0.07 kg^–1^. Energy demand associated with biomass drying for 72 h before and 24 h after pretreatment at 60 °C, followed by two-step pyrolysis for 1 h at 215 °C and 1 h at 400 °C, was estimated at 11.8–17.6 kWh kg^–1^ of biochar, corresponding to USD 1.77–2.64 kg^–1^.

The combined cost of these direct inputs was therefore estimated at USD 1.93–2.89 per kilogram of as-produced BC(215–400)­M. This value should not be interpreted as the total production cost because it excludes biomass logistics, labor, equipment depreciation, size reduction, process heat losses, emissions control, postproduction washing, wastewater treatment, and end-of-life management. Moreover, the 68% yield refers to the unwashed product and may include residual inorganic constituents retained after NaOH pretreatment.

Reported prices for commercial activated carbon provide only a broad economic reference and are not directly comparable with this partial production-cost estimate. Because no commercial activated carbon was evaluated under the same adsorption conditions, the present results do not establish the economic competitiveness of BC(215–400)­M on a treatment-performance basis. Such an assessment would require direct comparison of adsorption rate, working capacity, adsorbent dose, regeneration, operational lifetime, and cost per unit mass of TC removed. Accordingly, the present estimate is intended only to identify the principal direct cost contributions and to support a future techno-economic assessment.

## 4 Conclusions

This study demonstrates that the pyrolysis pathway strongly influences the physicochemical properties and adsorption behavior of biochars derived from NaOH-treated banana pseudostem. Among the unwashed materials, the two-step pathway produced BC(215–400)­M, which exhibited a more developed pore structure, greater accessibility of adsorption sites, and higher TC adsorption capacity than directly heated BC(400)­M. Under buffered conditions, BC(215–400)­M exhibited an adsorption capacity of 45.9 mg g^–1^ and reached equilibrium within approximately 12 h, whereas BC(400)­M required approximately 18 h. The better fit of the Freundlich model was consistent with adsorption on an energetically heterogeneous surface.

The apparent capacity of 177.1 mg g^–1^ obtained for unwashed BC(215–400)­M under unbuffered conditions highlights the strong influence of biochar-induced pH changes and residual inorganic species and should not be interpreted as an intrinsic material property. Postpyrolysis washing reduced its adsorption capacity by 11.0%, but the washed two-step biochar still outperformed its directly pyrolyzed counterpart by approximately 68%, confirming that its enhanced performance cannot be explained solely by soluble inorganic species. The results collectively suggest contributions from surface charge and functionality, aromatic domains, and pore accessibility, although individual adsorption mechanisms could not be independently confirmed. BC(215–400)­M retained substantial performance during two adsorption–desorption cycles and showed lower sensitivity to the tap water matrix than BC(400)­M, but its marked decline in the third cycle and the release of inorganic species identify regeneration and leaching control as priorities for further development. Overall, coupling NaOH pretreatment with two-step pyrolysis at a maximum temperature of 400 °C provides a promising route for valorizing banana pseudostem into TC adsorbents, while validation in actual wastewaters and optimization of end-of-life management remain necessary for practical implementation.

## Supplementary Material



## Notes

Additional data and discussion supporting the results presented in this manuscript, including complementary experimental results, characterization data, and further analysis and discussion of the findings (PDF)
