# Supplementary material for: Production of NaOH-Modified Banana Pseudostem Biochars through Controlled Heating for Tetracycline Removal
Source: ACS Omega. 2026 Oct 7;11(41):63276–98. doi: 10.1021/acsomega.6c05829 (PMC13689113; doi:10.1021/acsomega.6c05829)
Supplement: Supplementary file 1 [file ao6c05829_si_001.pdf]

Supporting Information

Production of NaOH-Modified Banana Pseudostem Biochars through Controlled Heating for Tetracycline Removal

Maria Elisa Avila Barboza[1], *João Antonio Tavares Barboza*[1], *Miriany Avelino Moreira Fernandez*[2], *and Guilherme Max Dias Ferreira*[1*]

[1]Group of Materials, Interfaces, and Solutions (MatIS), Department of Chemistry, Institute of Natural Sciences, Federal University of Lavras, Campus Universitário, Postal Code 37203-202, Lavras - MG, Brazil.
[2]Department of Chemistry, Institute of Natural Sciences, Federal University of Lavras, Campus Universitário, Postal Code 37203-202, Lavras - MG, Brazil.

KEYWORDS: Emerging contaminants; Agricultural residue; Alkaline activation; Water contamination; Heating Step

*Corresponding author
Name: Guilherme Max Dias Ferreira
Telephone: (+55) 35 3829-9797
E-mail: guilherme.max@ufla.br

Figures

Figure S1. Schematic representation of biomass modification and biochar production from banana pseudostem biomass by pyrolysis.

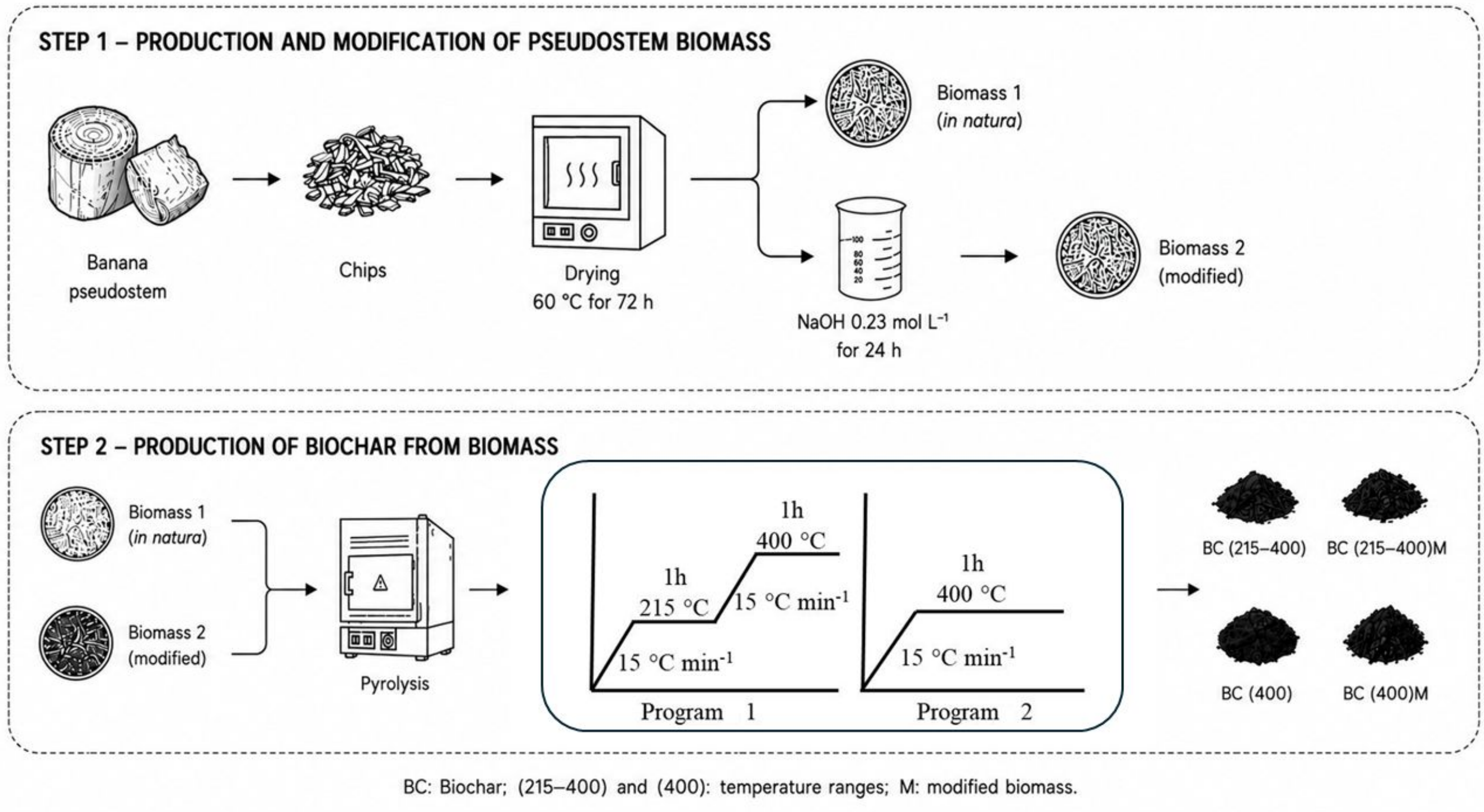

Figure S2. Micrographs obtained by scanning electron microscopy related to untreated banana pseudo-steam.

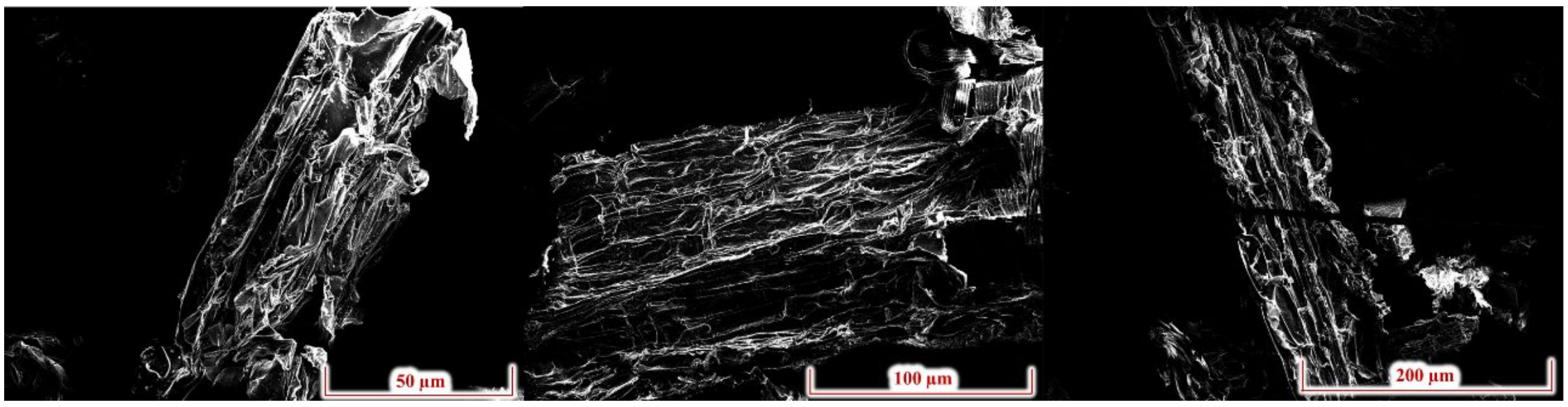

Figure S3. Micrographs obtained by scanning electron microscopy related to BC(400).

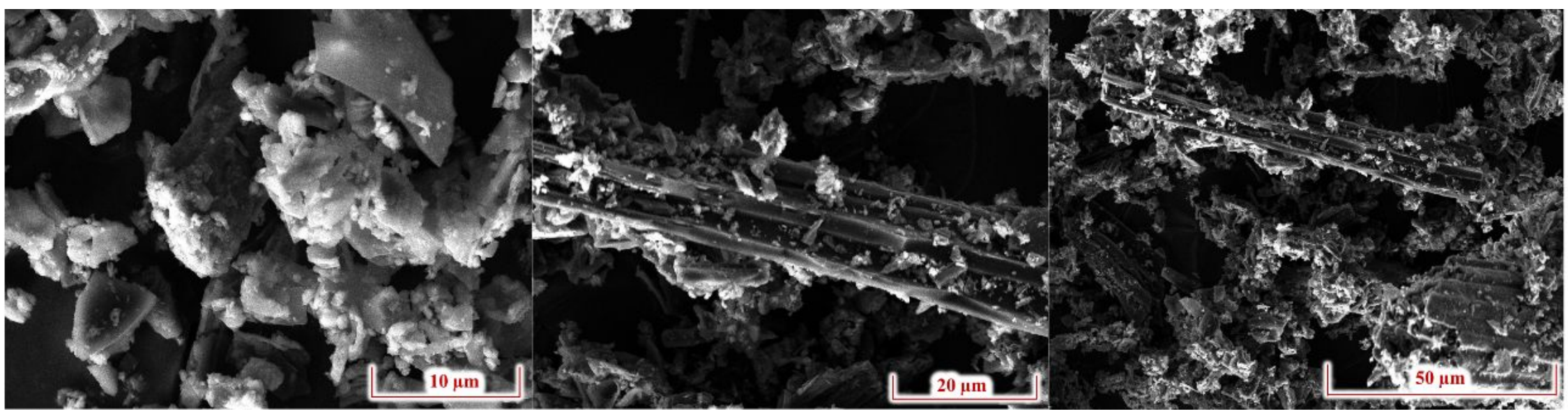

Figure S4. Micrographs obtained by scanning electron microscopy related to BC(215-400).

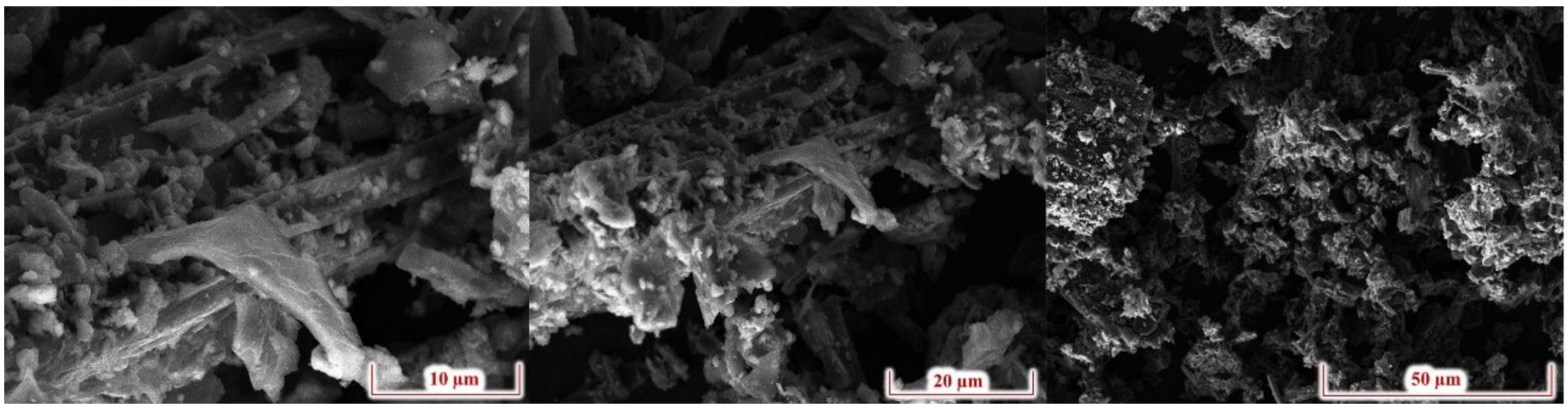

Figure S5. Micrographs obtained by scanning electron microscopy related to BC(400)M.

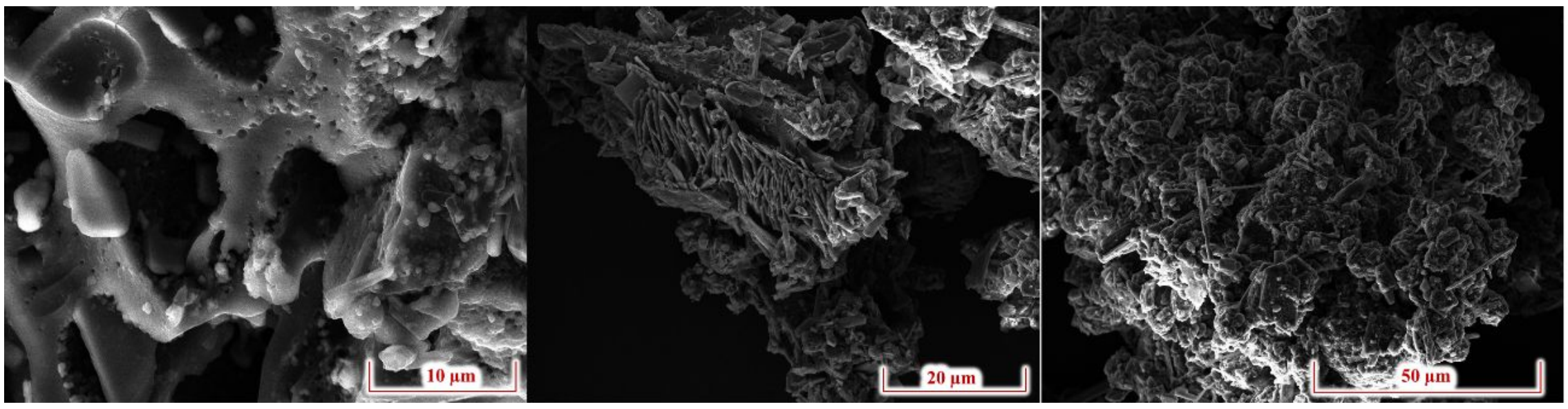

Figure S6. Micrographs obtained by scanning electron microscopy related to BC(215-400)M.

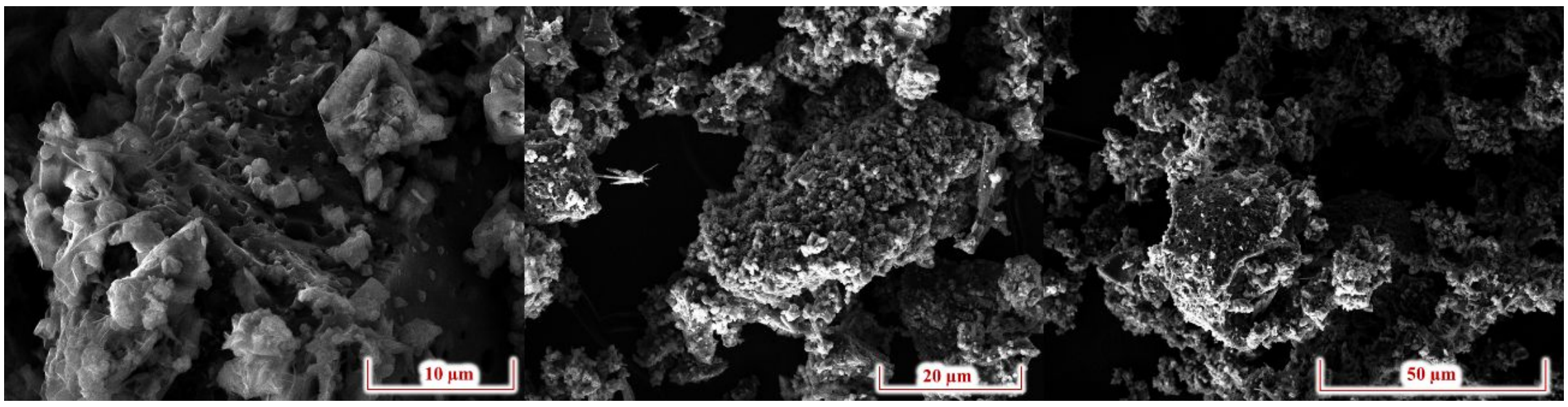

Figure S7. (a) FTIR spectra of washed samples BW(400) and BW(215-400)M. (b) X-ray diffraction (XRD) patterns of BC(400)M, BC(215-400)M, BW(400)M, and BW(215-400)M. The BC samples were analyzed under the same conditions for comparison. The broad amorphous signal in XRD is mainly attributed to the adhesive used to fix the samples and cannot be directly assigned to the biochars.

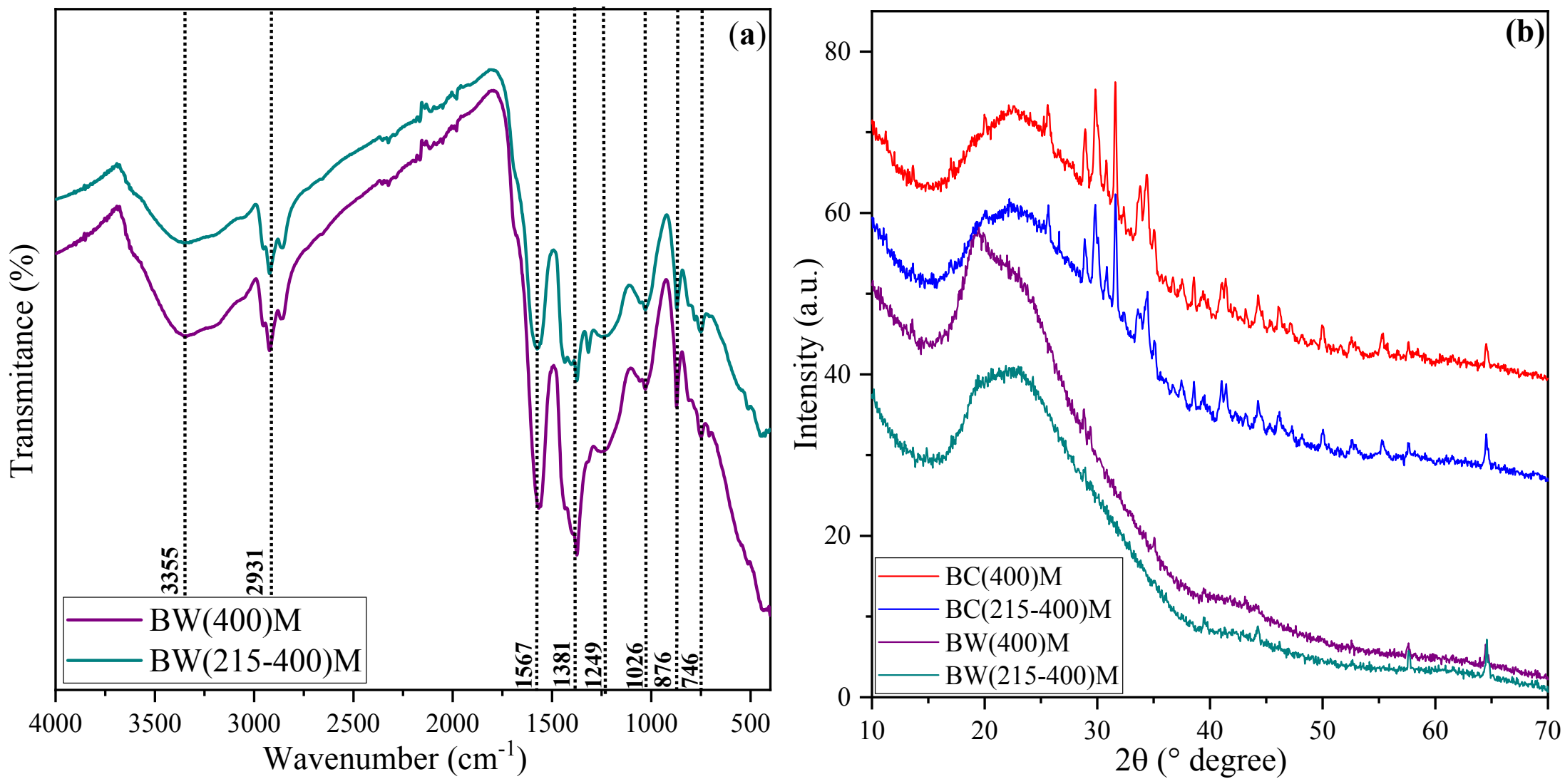

Characterization of the washed biochars provided additional information on the changes produced by washing. BW(400)M and BW(215–400)M retained the overall FTIR profiles of their corresponding unwashed materials (Figure S7a), although changes in band intensity were observed, particularly in the 1420–1450 cm^{-1} region associated with carbonate species (Figure S6). The persistence of the bands assigned to aromatic C=C stretching at approximately 1580–1600 cm^{-1} and C–O stretching at 1000–1200 cm^{-1} is consistent with preservation of the main carbonaceous surface functionalities. Thus, within the sensitivity of FTIR, washing did not produce substantial changes in the overall organic framework but affected carbonate-associated environments. The XRD patterns of the washed biochars (Figure S7b), obtained under the same conditions as those of the unwashed materials, showed a substantial decrease in the number and intensity of crystalline reflections. In particular, the attenuation or disappearance of reflections assigned to trona was consistent with the dissolution and removal of this readily soluble phase. Nevertheless, the absence of a diffraction peak after washing indicates that the corresponding phase was either removed or reduced below the XRD detection limit and does not demonstrate complete removal. The amorphous contribution was not interpreted because the adhesive used to mount the samples produced an intense broad signal. Collectively, the FTIR, XRD, and leaching results indicate that washing preferentially affected water-removable crystalline inorganic constituents while largely preserving the principal spectroscopically detectable features of the carbonaceous matrix.

Figure S8. Nitrogen adsorption-desorption isotherms of the biochars: (a) BC400 (b) BC (215–400) (c) BC (400)M and (d) BC (215–400)M.

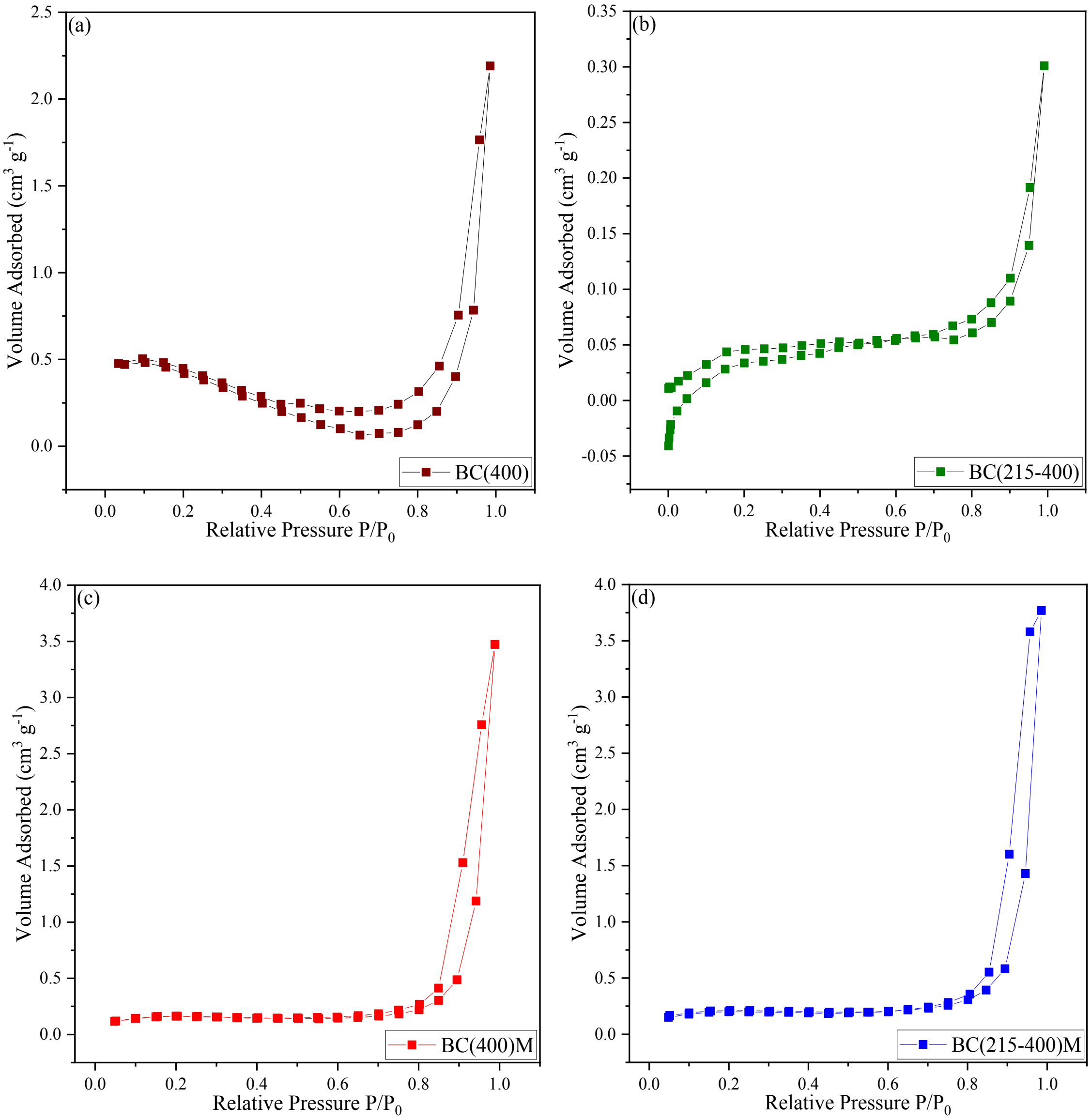

Figure S9. Determination of the point of zero charge (pH_{PZC}) of the biochars.

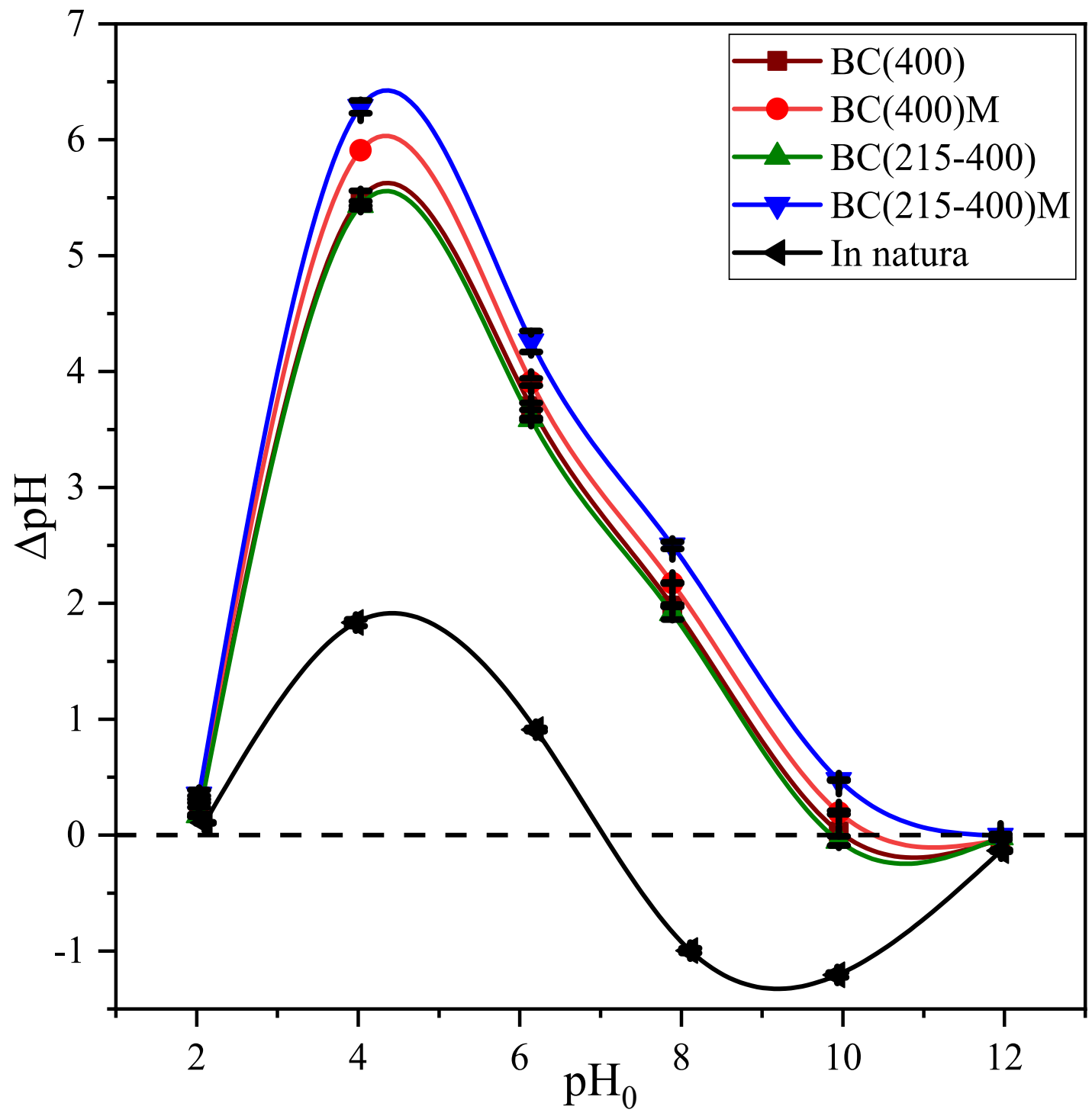

Figure S10. Conductometric titration curves of the materials for the determination of acidic and basic surface functional groups (a) BC 400 (b) BC 215-400 (c) BC 400M (d) BC 215-400M.

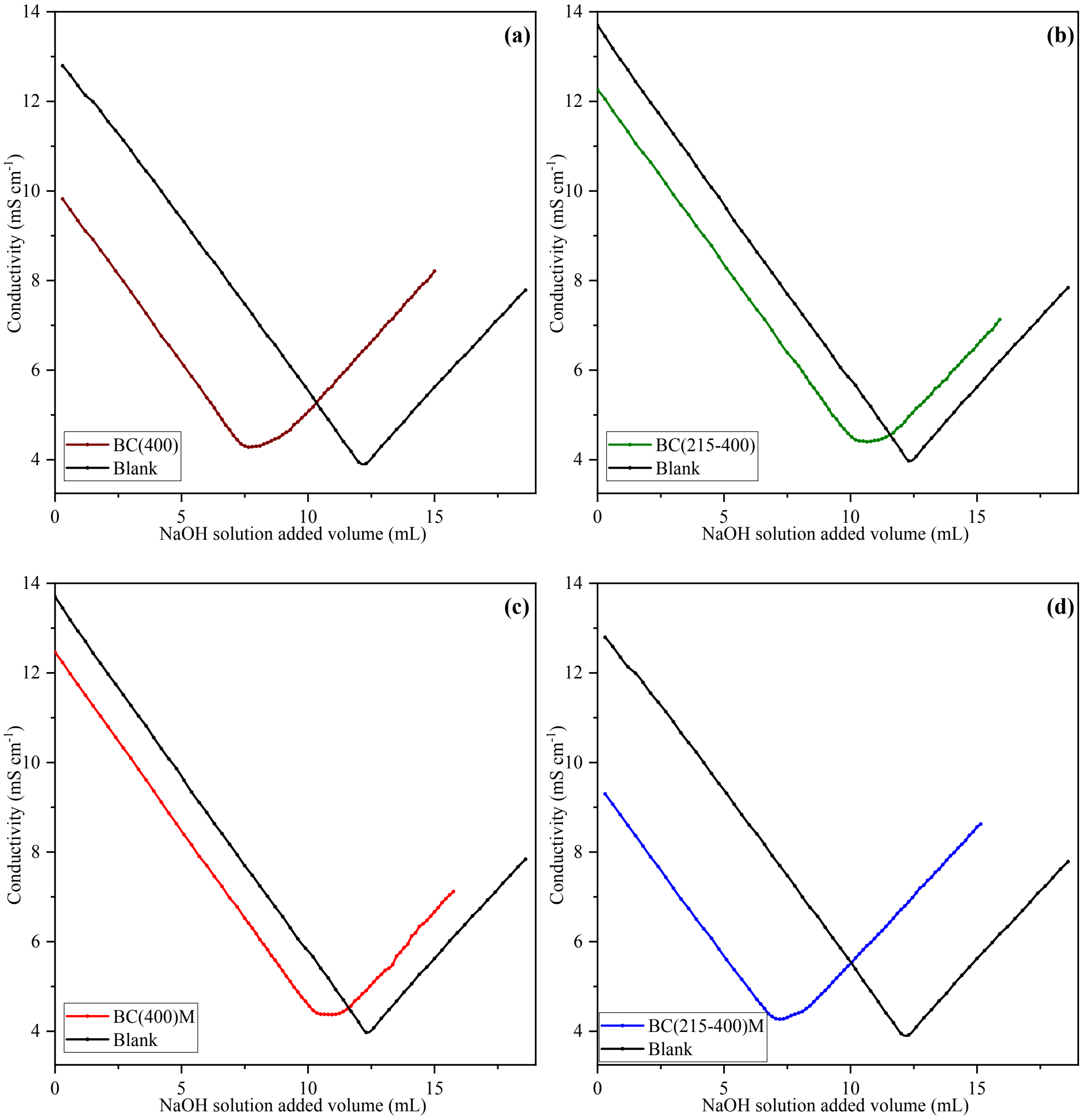

Figure S11. Preliminary performance tests of TC on biochars.

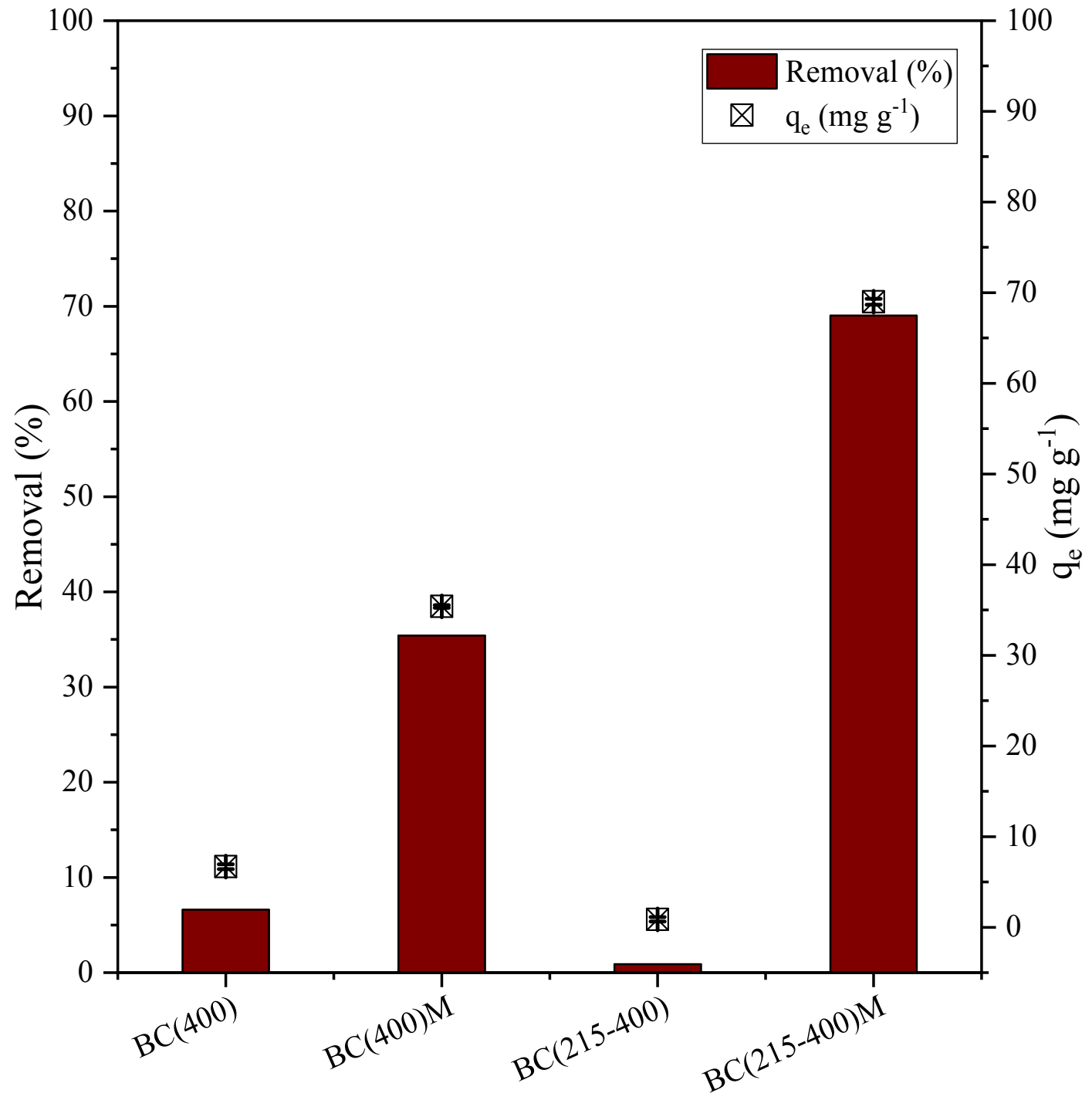

Conditions: initial concentration = 50 mg L^{-1}; pH = 6.0; adsorbent dosage 1.0 g L^{-1}; temperature 25 °C and 120 rpm for 24h. Error bars represent the standard deviation of three independent experiments (n = 3).

Figure S12. UV-Vis absorption spectra of TC at different pH values and shift in the maximum absorption as a function of pH. Conditions: Concentration of TC = 25 mg L^{-1}.

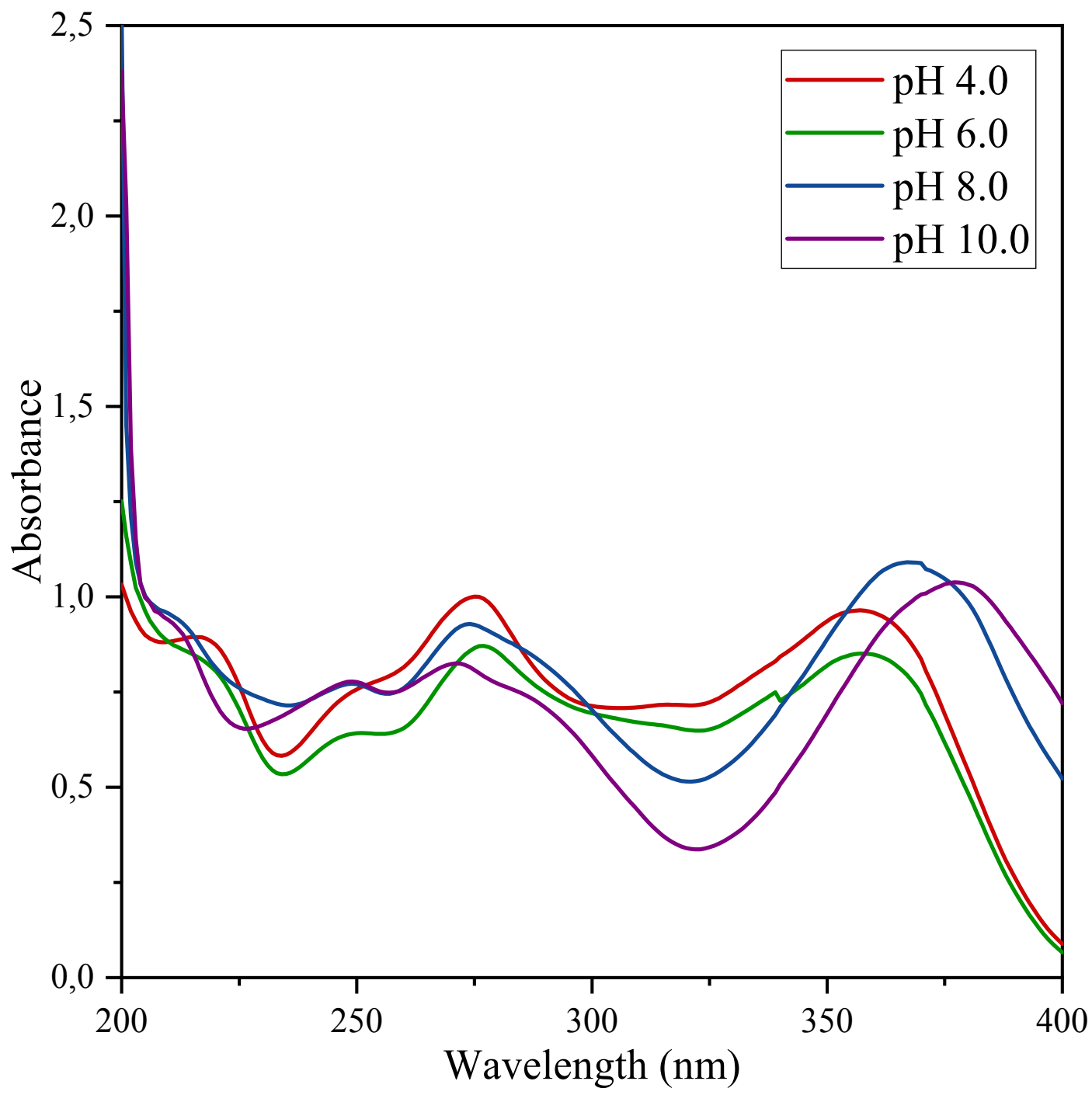

Figure S13. TG and DTG curves of BC(215-400)M after contact with deionized water (BC(215-400)M + DI) and after TC adsorption (BC(215-400)M + TC). Conditions: initial TC concentration = 400 mg L^{-1}; pH = 6.0; adsorbent dosage 1.0 g L^{-1}; temperature 25 °C and 120 rpm for 24h. Both samples were subjected to identical adsorption, washing, filtration, and drying procedures, except for the presence of TC in the adsorption system.

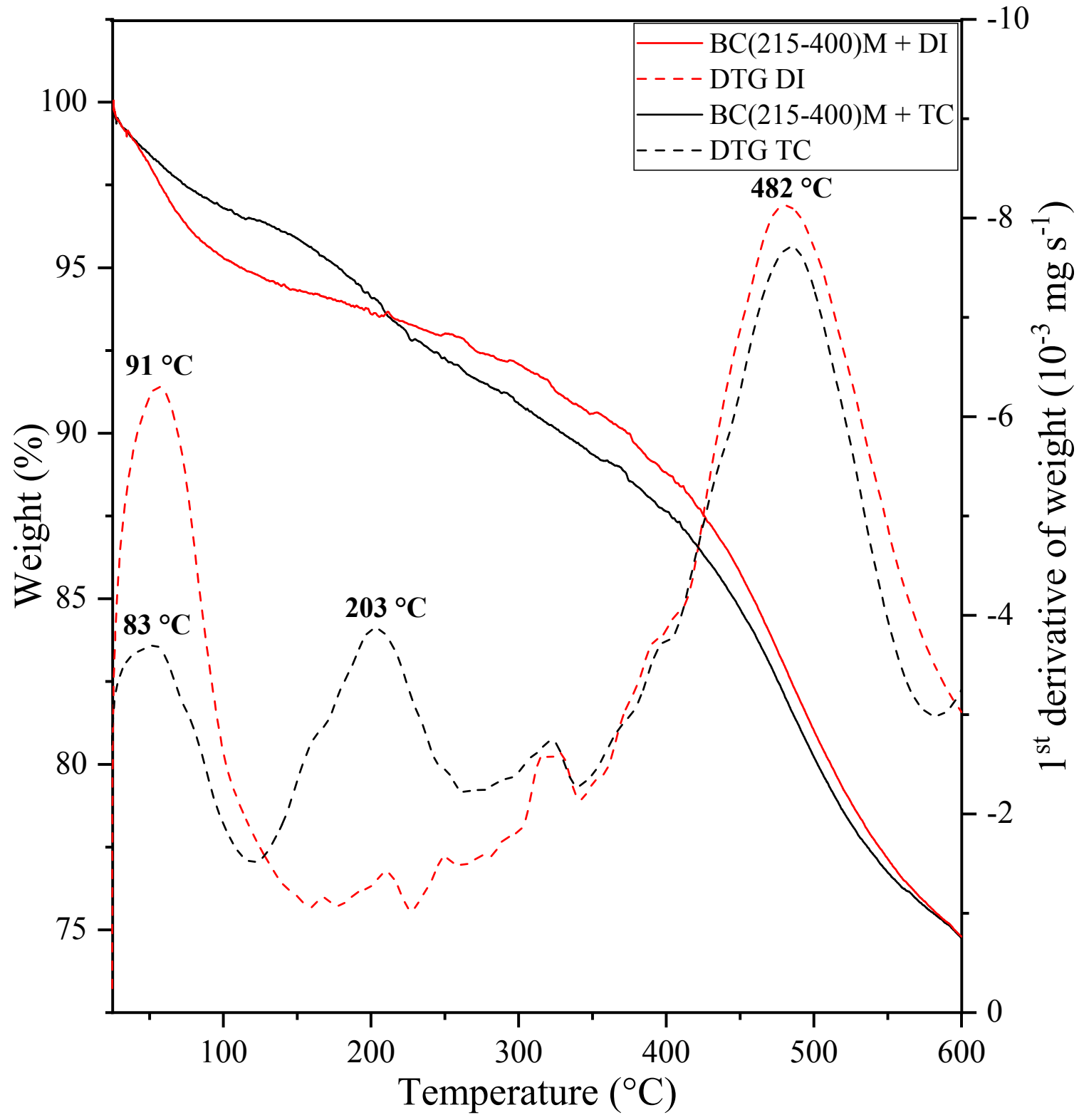

Figure S14. Reusability of BC(400)M and BC(215-400)M during successive adsorption–desorption cycles. Conditions: initial TC concentration = 50 mg L^{-1}; pH = 6.0; adsorbent dosage 1.0 g L^{-1}; temperature 25 °C and 120 rpm for 24h. Desorption was performed using 99.8% ethanol. Error bars represent the standard deviation of three independent experiments (n = 3).

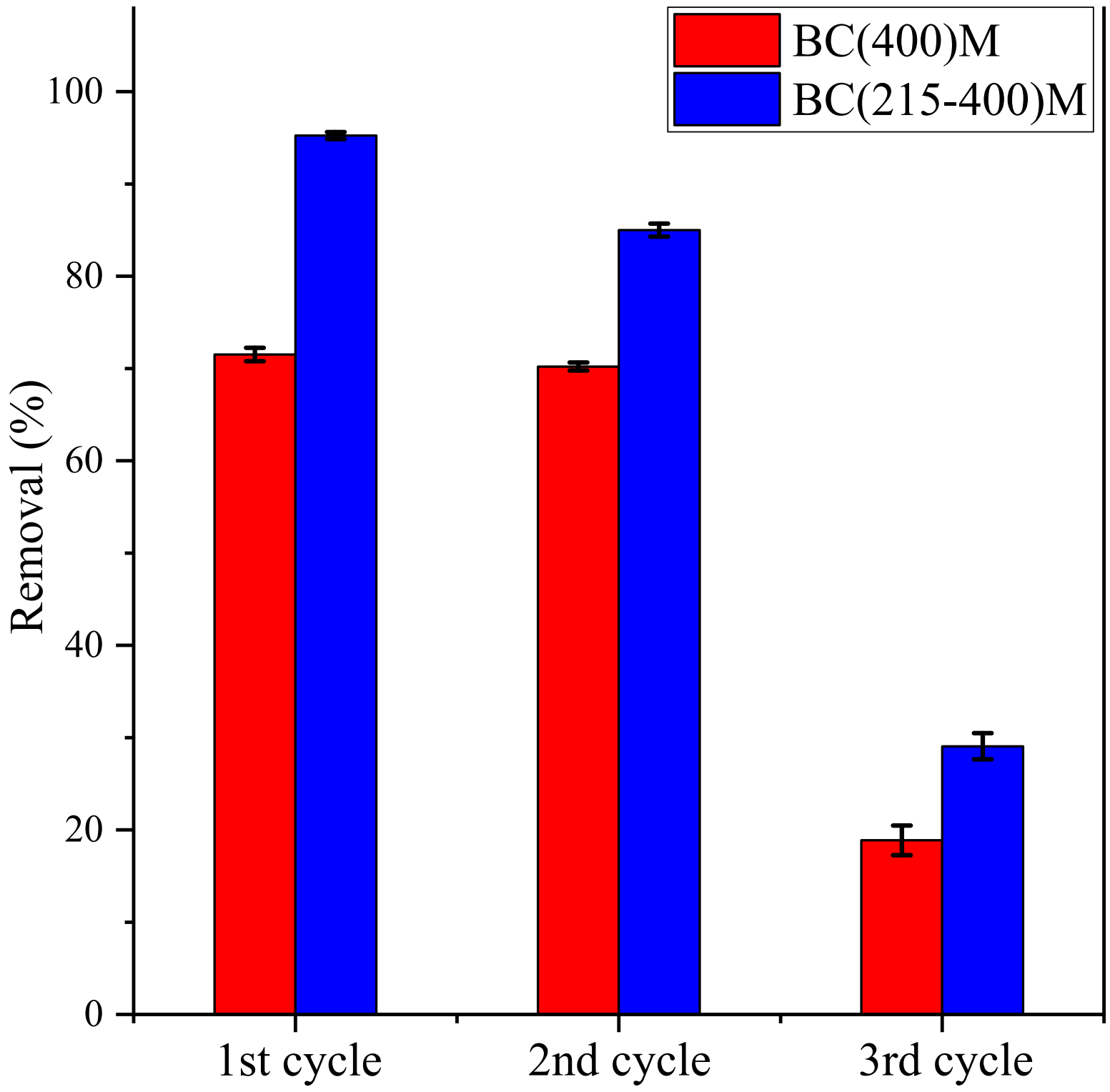

Tables

Table S1. Elemental composition of the samples obtained by energy-dispersive X-ray spectroscopy (EDS).

	Massa (%)								
**Sample**	O	K	Ca	Cl	Si	Al	Mg	Na	F
**BC 400**	18.2	7.5	2.0	0.5	0.2	0.1	0.5	-	2.1
**BC 400M**	31.0	11.0	0.8	1.5	-	-	-	12.9	-
**BC 215-400**	22.8	11.1	3.6	1.0	0.3	0.2	0.5	-	-
**BC 215-400M**	32.0	16.0	2.2	1.2	-	-	-	7.4	-

Table S2. pH_{PCZ} values and surface acidic and basic functional groups of the biochars.

**Sample**	**pH_{PCZ}**	**n_{acid} (mmol g^{-1})**	**n_{basic} (mmol g^{-1})**	**n_{total} (mmol g^{-1})**
**In natura**	7.1	-	-	-
**BC 400**	10.0	2.6	2.9	5.5
**BC 400M**	10.4	2.4	6.8	9.2
**BC 215-400**	9.9	2.4	2.9	5.3
**BC 215-400M**	11.7	1.7	6.8	8.5

Table S3. Maximum absorption wavelength at different pH values for TC.

**pH**	**Wavelength (nm)**	
4.0	275	356
6.0	276	357
8.0	274	367
10.0	271	377

Table S4. Final solution pH after the adsorption experiments. Initial pH was 6.0 for all experiments, except for pH effect on TC adsorption. Biochar dose = 1.0 g L^{-1}, temperature = 25°C, stirring at 120 rpm; and contact time of 24 h

**Experiment (Section in manuscript)**	**Condition***	**BC(400)M**	**BC(215-400)M**	**BW(400)M**	**BW(215-400)M**
Initial adsorption tests (Section 3.7 and 3.13)	A	10.11 ± 0.07	10.15 ± 0.08	7.5 ± 0.7	8.4 ± 0.5
	B	6.70 ± 0.06	6.85 ± 0.01	6.41 ± 0.08	6.16 ± 0.01
Effect of pH (Section 3.8)	C(2.03)	2.25 ± 0.02	2.23 ± 0.02	-	-
	C(4.08)	4.82 ± 0.02	4.78 ± 0.01	-	-
	C(6.00)	6.70 ± 0.06	6.85 ± 0.01	-	-
	C(8.04)	8.56 ± 0.02	8.58 ± 0.01	-	-
	C(10.06)	10.34 ± 0.02	10.44 ± 0.02	-	-
Electrolyte type (Section 3.10)	D	10.05	10.38	8.9	9.29
	E($CaCl_2$)	9.8 ± 0.4	10.3 ± 0.1	-	-
	E($MgSO_4$)	9.7 ± 0.4	10.7 ± 0.2	-	-
	E(KH_2PO_4)	9.8 ± 0.3	10.3 ± 0.2	-	-
Tap water (Section 3.11)	F	8.18 ± 0.1	8.6 ± 0.1	-	-
Reuse (Section 3.15)	G(Cycle 1)	10.17 ± 0.04	10.26 ± 0.05	-	-
	G(Cycle 2)	10.09 ± 0.05	10.3 ± 0.1	-	-
	G(Cycle 3)	9.87 ± 0.03	10.07 ± 0.02	-	-

*A = 100 mg L^{-1} TC in deionized water; B = 100 mg L^{-1} TC in 0.01 mol L^{-1} B–R buffer; C = 50.0 mg L^{-1} TC in 0.01 mol L^{-1} B–R buffer at different initial pH values, indicated in parentheses; D = deionized water without TC; E = 100 mg L^{-1} TC in 0.1 mol L^{-1} salt solutions without buffer; F = 100 mg L^{-1} TC in tap water without buffer; and G = 50 mg L^{-1} TC in deionized water during successive adsorption–desorption cycles.
